# PreCurious: How Innocent Pre-Trained Language Models Turn into Privacy Traps

**DOI:** 10.1145/3658644.3690279

**Published:** 2024-12-09

**Authors:** Ruixuan Liu, Tianhao Wang, Yang Cao, Li Xiong

**Affiliations:** Emory University, Atlanta, USA; University of Virginia, Charlottesville, USA; Tokyo Institute of Technology, Tokyo, Japan; Emory University, Atlanta, USA

**Keywords:** Privacy Attack, Language Model, Pre-Training

## Abstract

The pre-training and fine-tuning paradigm has demonstrated its effectiveness and has become the standard approach for tailoring language models to various tasks. Currently, community-based platforms offer easy access to various pre-trained models, as anyone can publish without strict validation processes. However, a released pre-trained model can be a privacy trap for fine-tuning datasets if it is carefully designed. In this work, we propose PreCurious framework to reveal the new attack surface where the attacker releases the pre-trained model and gets a black-box access to the final fine-tuned model. PreCurious aims to escalate the general privacy risk of both membership inference and data extraction on the fine-tuning dataset. The key intuition behind PreCurious is to manipulate the memorization stage of the pre-trained model and guide fine-tuning with a seemingly legitimate configuration. While empirical and theoretical evidence suggests that parameter-efficient and differentially private fine-tuning techniques can defend against privacy attacks on a fine-tuned model, PreCurious demonstrates the possibility of breaking up this invulnerability in a stealthy manner compared to fine-tuning on a benign pre-trained model. While DP provides some mitigation for membership inference attack, by further leveraging a sanitized dataset, PreCurious demonstrates potential vulnerabilities for targeted data extraction even under differentially private tuning with a strict privacy budget e.g. ϵ=0.05. Thus, PreCurious raises warnings for users on the potential risks of downloading pre-trained models from unknown sources, relying solely on tutorials or common-sense defenses, and releasing sanitized datasets even after perfect scrubbing.

## 1 Introduction

The pre-training and fine-tuning paradigm has become the standard approach for tailoring language models (LMs) to various tasks, such as the medical domain [15, 22]. In this approach, a language model is pre-trained on a large, general dataset and then fine-tuned on a smaller, domain-specific dataset. Privacy risks arise when the fine-tuning data is private and the fine-tuned model can be accessed as a service [34]. One realistic scenario is that a hospital fine-tunes a model using local Electronic Health Record (EHR) data and then shares the API with other hospitals that lack such expertise. Existing works broadly explore the privacy risks of training data via black-box access of the model [3, 6, 41], which is also applicable to the fine-tuned model.

In this paper, we reveal a new privacy attack surface where an attacker aims to escalate the privacy risk of the fine-tuning data from a fine-tuned model by manipulating the pre-trained language model loaded by the user before fine-tuning and then getting the black-box access to the fine-tuned model. This is realistic since anyone can publish models on community-based platforms (e.g., Huggingface [2], GitHub [1]) without stringent validation processes. A fine-tuning user may inadvertently download an untrusted pretrained model from compromised sources, especially when popular models have different variants on platforms like Hugging Face. For instance, a victim could make a typo during the download process or fall for a malicious higher-version package registered with the same name as a legitimate model.

Previous work [44] explored additional adversarial access besides the black box access of the model by injecting poisoned data in the training dataset to amplify the privacy risk, which requires the adversarial capability of accessing/crafting training data. A recent work [43] manipulates pre-trained (upstream) image classification model for increasing the privacy risk of downstream models, but is limited to property inference attacks that infer whether images with a specific attribute are used for training. In our threat model, the attacker aims to escalate the privacy risk by manipulating the released pre-trained model, without assuming access to the fine-tuning process or fine-tuning dataset. Our adversarial goal is to amplify fundamental privacy threats of membership inference attack [3] and data extraction [6] in the fine-tuned language model, compared to the one fine-tuned from a benign pre-trained model.

**Figure 1:**
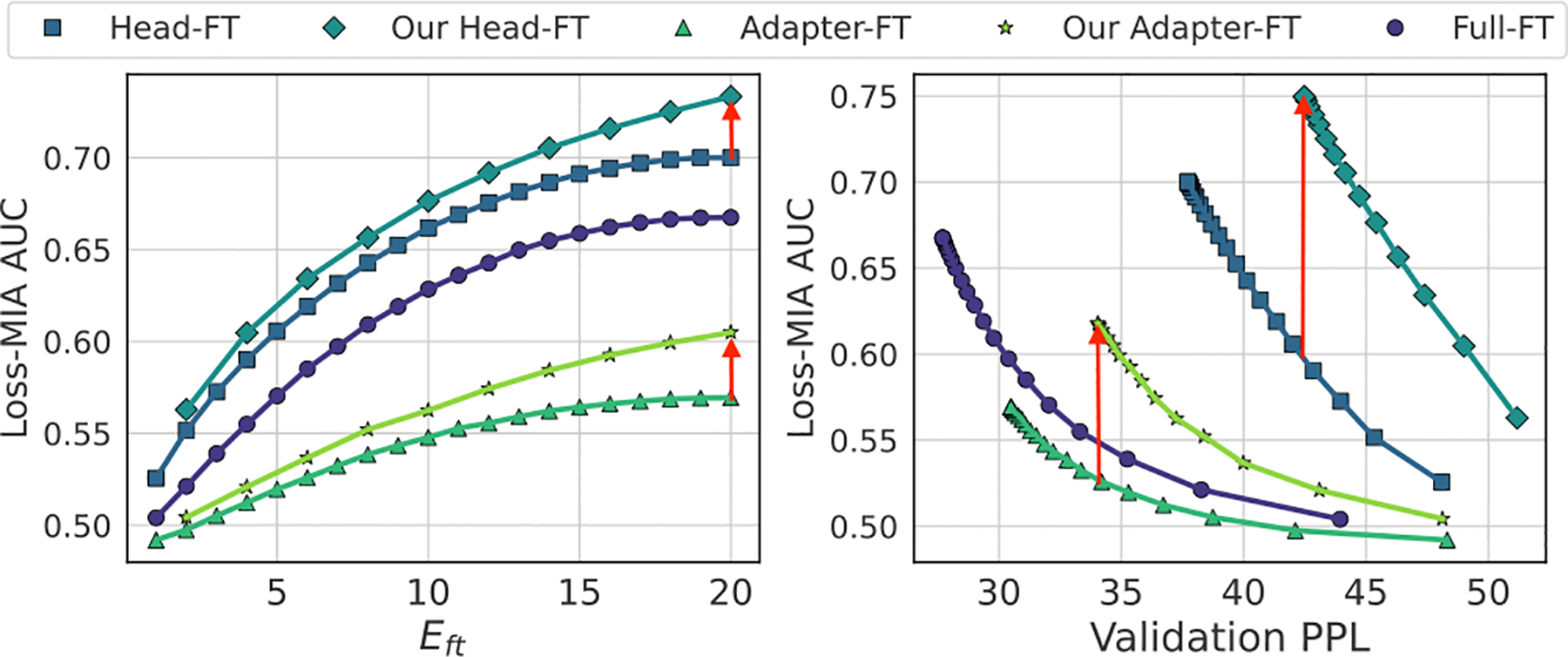
The privacy vulnerability for target models fine-tuned by various methods ranks as Head-FT > Full > Adapter-FT. *PreCurious* increases the privacy risk for each iteration and ruins the privacy-utility trade-off, as demonstrated with Head-FT and Adapter-FT. $E_{\text{ft}}$ indicates the fine-tuning epochs and lower validation perplexity means better performance.

It is non-trivial to achieve our privacy risk amplification goal since parameter-efficient fine-tuning (PEFT) techniques such as Adapter [37] and LoRA [14] have been established to have a privacy invulnerability property [34, 46]. This is demonstrated in Figure 1 which shows the privacy vulnerability (measured in membership inference attack (MIA) effectiveness in AUC) for different fine-tuning methods vs. the fine-tuning epochs (left) and utility of the fine-tuned model (right) (measured in validation perplexity (PPL)). We can see that the Adaptor fine-tuning (Adaptor-FT) exhibit a very low vulnerability. At the same time, the training efficiency introduced by PEFT [12, 26] makes it broadly applicable for LMs, especially encouraging differentially private (DP) fine-tuning for a large model [24], which makes the privacy attacks on the fine-tuned model more challenging.

**Figure 2:**
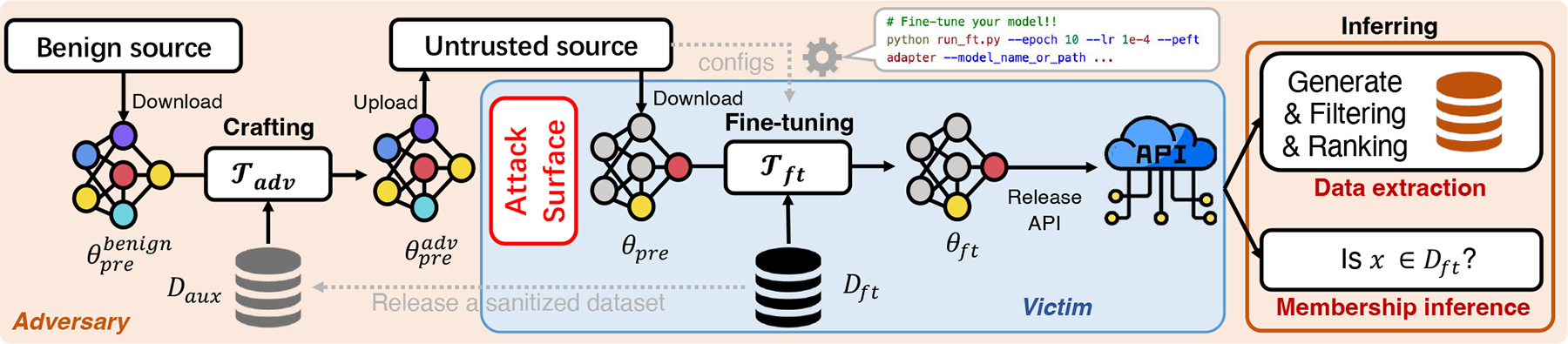
Framework overview of *PreCurious*. The dashed gray line indicates extra side information that can be utilized: 1) the stopping criterion, 2) the fine-tuning method, and 3) the released sanitized data by masking the secret. We design *Accelerated* and *Lagging* strategies for stopping by epoch or by performance. We propose an aggressive anti-freezing strategy when the victim uses the given fine-tuning method. We utilize a released sanitized dataset in targeted data extraction experiments.

Our key intuition is to manipulate the memorization level of the pre-trained model by exploiting PEFT. Since the majority of the pre-trained model is frozen during PEFT, we can better influence the behavior of the trainable modules for amplifying risks in the fine-tuned model. Figure 2 illustrates our proposed framework where an attacker downloads a benign large model, manipulates it by an auxiliary dataset, and uploads it to an untrusted source for victims. We exploit side information such as the stopping criterion and the fine-tuning method by implicitly guiding the fine-tuning victims through documents or tutorials, proposing *lagging* or *accelerating* strategies for cases with or without early stopping and *anti-freezing* strategy when fine-tuning method is known. Additionally, we attempt to make full use of the public information, for example, a released de-identified dataset, to further enhance the attack capability.

We demonstrate that our attack can successfully amplify various privacy risks. Figure 1 illustrates the increased privacy vulnerability of MIA by our methods on both Head-FT and Adaptor-FT. More generally, for MIA, we compare PreCurious with benign GPT-2 [38] on the same black-box attack and demonstrate that by manipulating the pre-trained model, the true-positive-rate (TPR) at a false-positive-rate (FPR) of 0.01% on Enron [21], PubMed [9] and PTB [31] datasets is boosted by 8×, 131× and 36×, respectively. For untargeted data extraction attack, we increase the times for a less duplicated sub-sequence shown in the pool of filtered generations by around 10×. For targeted data extraction attack on Enron dataset, fine-tuning over benign model initialization cannot expose any secrets when fine-tuning with a strong DP level (ϵ=0.05) while *PreCurious* can extract 3 target email addresses with valid exposure values. As advocated by previous work [44], we also audit the stealthiness of *PreCurious* and propose a mitigation method to make it more stealthy.

Our contribution can be summarized as follows:

- We propose a framework *PreCurious* to amplify the privacy risk of both membership inference and data extraction in the pre-training and fine-tuning paradigm, revealing the risk of fine-tuning over an unofficially released pre-trained LM.
- We propose two memorization manipulating strategies to craft the pre-trained model for fine-tuning with or without early-stopping. We further exploit the side-information of PEFT or sanitized dataset to enhance the attack effectiveness.
- We demonstrate the underestimated vulnerability of commonsense defenses, including regularization, differentially private fine-tuning, and deduplication with *PreCurious*, particularly highlighting risks for users who rely on common-sense defenses without auditing privacy and training dynamics.
- We demonstrate the risks of publishing de-identified datasets solely by removing personally identifiable information (PII), as *PreCurious* can exploit the context to extract targeted secrets if the original datasets are involved in future fine-tuning, underscoring significant vulnerabilities in the data release.

## 2 Threat Model and Preliminaries

We formulate the threat model and preliminaries in this section. The attack framework of *PreCurious* sits in the pre-training and fine-tuning paradigm of language models (LMs) to amplify data leakage in the fine-tuning stage.

Our target victim model is fine-tuned with the basic next-token prediction task. The model aims to predict the next token $x_{t}$ given the previous tokens $\left(x_{1},x_{2},\ldots ,x_{t-1}\right)$ for a given text sequence with T tokens. The fine-tuning involves minimizing the objective: $ℒ=-\sum_{t=1}^{T}\text{log}\;f_{\theta }\left(x_{t}|x_{i<t}\right)$, where $f_{\theta }\left(x_{t}|x_{i<t}\right)$ is the probability of $x_{t}$ from the softmax output of the model θ. The trained model can generate new text by iteratively sampling ${\overset{ˆ}{x}}_{t}\sim f_{\theta }\left(x_{t}|x_{i<t}\right)$ and feeding it to sample the next token.

### 2.1 Parameter-Efficient Fine-tuning (PEFT)

Denoting the fine-tuned model as $\theta_{\text{ft}}=\theta_{\text{pre}}\circ \Phi$, the key idea of PEFT is only optimizing over small modules Φ while freezing $\theta_{\text{pre}}$, which transfers the fine-tuning objective as $ℒ=-\sum_{t=1}^{T}\text{log}\;f_{\Phi }\left(x_{t}|x_{i<t},\theta_{\text{pre}}\right)$.

One line of *selective* PEFT selects a portion of parameters in $\theta_{\text{pre}}$ as Φ, such as Head-FT with a few top layers [10] and Bitfit-FT with the bias terms of the model [52]. The other line of PEFT introduces new randomly initialized modules as Φ as plug-in for $\theta_{\text{pre}}$. For example, *additive* method Adapter-FT [13] inserts small and trainable fully connected networks Φ after transformer sub-layers in $\theta_{\text{pre}}$. The *reparameterization-based* method LoRA-FT [14] employs a low-rank matrix decomposition to parameterize the weight updates, and Φ indicates parameters for the low-rank matrices.

### 2.2 Threat Model

*PreCurious* indicates the pre-trained model releaser is curious about the private fine-tuning dataset $D_{\text{ft}}\in 𝒟$. We consider the model fine-tuner as the challenger C (or victim), and pre-trained model publisher as the adversary 𝒜.

#### 2.2.1 Adversarial Capabilities.

We make two common adversarial capability assumptions. First, we follow a common assumption [32, 39, 48, 50] that the adversary can query the loss value for a given sample via black-box access. Second, following previous works [17, 34, 39, 41, 44, 45, 48], we assume the adversary has an auxiliary dataset $D_{\text{aux}}\in 𝒟$ drawn from the same distribution but disjoint from the fine-tuning dataset $D_{\text{ft}}$. Different from capabilities in backdoor attacks on the pre-trained model, we do not assume either access to pre-training dataset of the original backbone [18] or the access to the samples in downstream dataset [53]. Additionally, we do not require capability of injecting poisoned data [44] or tampering the fine-tuning process.

Distinguished from all existing works, the adversary in *PreCurious* releases the pre-trained model with seemingly legitimate configuration documents, which is very common when sharing customized models on open-sourced platforms. We also note that even for popular pre-trained models, the victim may inadvertently download an untrusted $\theta_{\text{pre}}$. In this case, attackers could use the official model’s default configuration in tutorials, which victims assume as correct. First, typographical errors during the search and download process, such as hf_hub_download(repo_id=NAME_WITH_TYPO) in Hugging Face, could lead to the acquisition of a malicious model. Second, attackers could register publicly available higher-version packages with the same name as the legitimate model, which could be automatically installed via library management tools. Finally, the attacker could compromise the repository’s infrastructure and replacing the legitimate pre-trained model with a malicious one.

The seemingly legitimate configuration $C=\left\{C_{\text{stop}},C_{\text{peft}}\right\}$ includes: 1) stopping criterion $C_{\text{stop}}\in \left\{c_{\text{epoch}},c_{\text{perf}}\right\}$ of stopping-by-epochs or early-stopping-by-performance without imposing fixed hyper-parameters, and 2) PEFT strategy $C_{\text{peft}}$ like Adapter-FT or LoRA-FT that can be easily set using open-source frameworks [37]. $C_{\text{peft}}$ is optional and only used for an accelerated variant in Section 3.2.2.

We do not require the adversarial capability to pre-train a language model from scratch. Thus, we assume the released pre-trained model $\theta_{\text{pre}}^{\text{adv}}$ is crafted from a benign model $\theta_{\text{pre}}^{\text{benign}}$ downloaded from a trusted source.

#### 2.2.2 Privacy Game.

Now we construct the general privacy game between a challenger C (the model fine-tuner) and an adversary 𝒜 (the pre-trained model publisher) in Game 1.

Game 1 (Privacy game in *PreCurious*).

- *The adversary crafts and releases model with a suggested configuration* $C,\;\theta_{\text{pre}}^{\text{adv}}\leftarrow {𝒯}_{\text{adv}}\left(D_{\text{aux}}|\theta_{\text{pre}}^{\text{benign}},C\right)$.
- *The challenger samples a training dataset* $D_{ft}\in D$ *and a secret* z∈𝒰 *(such that $D_{ft}\cap 𝒰=\emptyset )$*, *combining as $D_{ft}\leftarrow D_{ft}\cup \{z\}$*
- *The challenger loads $\theta_{\text{pre}}^{\text{adv}}$* as the model initialization, follows C *in fine-tuning and releases the black-box access to the final model* $\theta_{ft}^{adv}\leftarrow {𝒯}_{ft}\left(D_{ft}|\theta_{pre}^{adv},C\right)$.
- *The adversary queries $\theta_{ft}^{\text{adv}}$ and emits a guess $\overset{ˆ}{z}\in 𝒰$*.
- *The adversary wins the game if* $\overset{ˆ}{z}=z$.

We use 𝒰 to denote the secret universe of $D_{\text{ft}}$. Removing the procedures in red and replacing $\theta_{\text{pre}}^{\text{adv}}$ with a benign model $\theta_{\text{pre}}^{\text{benign}}$ reduces Game 1 to a conventional privacy game.

#### 2.2.3 Adversarial Goal.

The adversary aims to increase the privacy risk in the fine-tuning training dataset $D_{\text{ft}}$. We focus on two representative privacy notions as follows:

- **Membership Priavcy** [41] is defined on the existence of a given sample in the fine-tuning dataset $D_{\text{ft}}$.
- **Extraction Privacy** [6] is defined on the verbatim extraction of a subsequence in $D_{\text{ft}}$. The extraction is targeted if the attacker defines the format of secrets before the attack.

Concretely, 𝒰 covers both membership privacy and extraction privacy by different instantiations. For example, for membership inference, 𝒰={x,⊥} denotes two cases where a sample x is or is not in $D_{\text{ft}}$. For data extraction, 𝒰 consists of the collection of all candidate secrets for a piece of text in $D_{\text{ft}}$.

Furthermore, the adversary aims to amplify the privacy risk in $D_{\text{ft}}$ compared to fine-tuning from a benign model, as formally defined in Definition 2.1.

Definition 2.1 (Successful privacy risk amplification). *Given the same fine-tuning procedure ${𝒯}_{ft}$ based on a benign model $\theta_{\text{pre}}^{\text{benign}}$*, *and considering two privacy games differentiated by ${𝒯}_{\text{adv}}$ as* $𝒢{≃}_{{𝒯}_{\text{adv}}}\;{𝒢}^{\prime }$, *the privacy risk is amplified by* ${𝒯}_{\text{adv}}$ *when the adversarial gain:*

$$\Delta \text{Ad}v_{𝒢{≃}_{{𝒯}_{\text{adv}}}{𝒢}^{\prime }}^{\text{ft}}=\text{Ad}v_{𝒢}\left(𝒜,D_{\text{ft}},\theta_{\text{ft}}^{\text{adv}},z|{𝒯}_{\text{adv}}\right)\;-\text{Ad}v_{{𝒢}^{\prime }}\left(𝒜,D_{\text{ft}},\theta_{\text{ft}}^{\text{benign}},z\right)>0.$$

The ${\text{Adv}}_{𝒢}(𝒜,\cdot )$ can be a success metric for reflecting the adversary’s advantage for a specific attack, for example, ${\text{Adv}}_{{𝒢}_{\text{MIA}}}(𝒜,\cdot )=2\cdot \text{Pr}[\overset{ˆ}{z}=z]-1$ for MIA [40].

Meanwhile, the adversary should avoid suspicions from victims that the pre-trained model $\theta_{\text{pre}}^{\text{adv}}$ will increase privacy risks in $D_{\text{ft}}$. As defined in Definition 2.2, we simulates the risk auditing based on the most ideal assumption for victims to have a benign model. Note that Definition 2.1 is computed on the fine-tuned model, while Definition 2.2 is measured on the pre-trained model.

Definition 2.2 (Privacy risk amplification stealthiness). *The pre-trained model $\theta_{\text{pre}}^{\text{adv}}$ output by a crafting algorithm* ${𝒯}_{\text{adv}}$ *is stealthy when the adversarial gain compared to* $\theta_{\text{pre}}^{\text{benign}}$ satisfies:

$$\Delta Adv_{𝒢≃{𝒯}_{adv}}^{\text{pre}}{𝒢}^{\prime }=Adv_{𝒢}\left(𝒜,D_{ft},\theta_{\text{pre}}^{\text{adv}},z|{𝒯}_{\text{adv}}\right)\;-Adv_{{𝒢}^{\prime }}\left(𝒜,D_{ft},\theta_{\text{pre}}^{\text{benign}},z\right)\approx 0.$$

For stealth, the simplest but most effective way is not involving any fine-tuning samples in the crafting phase, which is consistent with the adversarial capabilities defined in Section 2.2.1 that 𝒜 knows no exact samples in $D_{\text{ft}}$ and $D_{\text{aux}}$ is disjoint from $D_{\text{ft}}$. As models cannot memorize secret before seeing it, the adversarial gain compared to the benign model for $D_{\text{ft}}$ should satisfy Definition 2.1.

### 2.3 Success Metrics

Now we introduce concrete attack effectiveness metrics for different attacks and propose stealthiness metrics for victims to audit the pre-trained model.

#### 2.3.1 Membership Inference Attack.

We use AUC ↑ to measure the effectiveness of the attack (↑ means the higher the value the more desirable the metric). As suggested by previous work [3], we also present results for MIA with TPR@FPRα% ↑ given a small α. A lower α emphasizes the cost of false positives.

#### 2.3.2 Data Extraction Attack.

For untargeted data extraction, we follow previous work [23] to capture the portion $p_{\text{ext}}$ ↑ of sub-sequences emitted by the target model that are included in the fine-tuning dataset $D_{\text{ft}}$. For targeted data extraction, we use the exposure [5] to measure if a targeted secret such as a phone number or email address can be reliably extracted.

#### 2.3.3 Stealthiness.

Following Definition 2.2, we propose three representative metrics as indicators of the adversarial gain, and the difference compared with a benign model reflects the stealthiness of the released model.

First, we simulate MIA with a non-membership dataset drawn from the same distribution to audit the stealthiness $S_{\text{mia}}$ by using MIA success metrics such as AUC in Section 2.3.1.

Second, for simulating the data extraction attack in an efficient way [4], we use the k-extractable rate as $S_{\text{mem}}=\frac{1}{n}\sum_{i}^{n}I_{k\text{-extract}}\left(x,\theta_{\text{pre}}\right)$, where $I_{k\text{-extract}}=1$ indicates if the model can generate the suffix s given a k-length prefix x=[p‖s].

Lastly, as overfitting is considered as an important cause of various privacy attacks, the victim may calculate the performance difference $S_{\text{gap}}$ between the training and validation dataset as a signal of overfitting: $S_{\text{gap}}=\text{PPL}\left(D_{\text{val}}|\theta_{\text{pre}}\right)-\text{PPL}\left(D_{\text{ft}}|\theta_{\text{pre}}\right)$, where PPL is a standard performance metric of LMs.

Assuming the benign model derives the baseline stealthiness metric $S_{\text{mia}}=0.5$ for AUC, $S_{\text{mem}}=0$, and $S_{\text{gap}}\leq 0$, our goal is to ensure a low gap for $\theta_{\text{pre}}^{\text{adv}}$ compared to the baseline.

## 3 Amplifying Privacy Risk with PreCurious

In this section, we introduce the *PreCurious* framework shown in Figure 2, crafting methodologies, and the inference pipelines.

### 3.1 Attack Overview

#### 3.1.1 PreCurious Framework.

We begin with a high-level overview of the pre-curious attack which involves the following three stages. 1) **Crafting**: the adversary carefully crafts the backbone model before releasing it as a pre-trained model. 2) **Fine-tuning**: the victim initializes the model with the released parameters and starts normal fine-tuning over the private training dataset. 3) **Inferring**: the adversary queries the target model and guesses secrets in $D_{\text{ft}}$.

*PreCurious* focuses on designing the crafting stage for increasing the attack advantage and thus stands as a general framework for a wide range of inferring strategies.

#### 3.1.2 Key Intuition.

From the feasible and limited capabilities in Section 2.2.1, we notice that the one more thing that 𝒜 can manipulate than a conventional attacker is the model initialization in the **crafting** stage. Thus, we can first focus on the design of the model initialization in crafting and keep a basic inferring phase for now.

**Figure 3:**
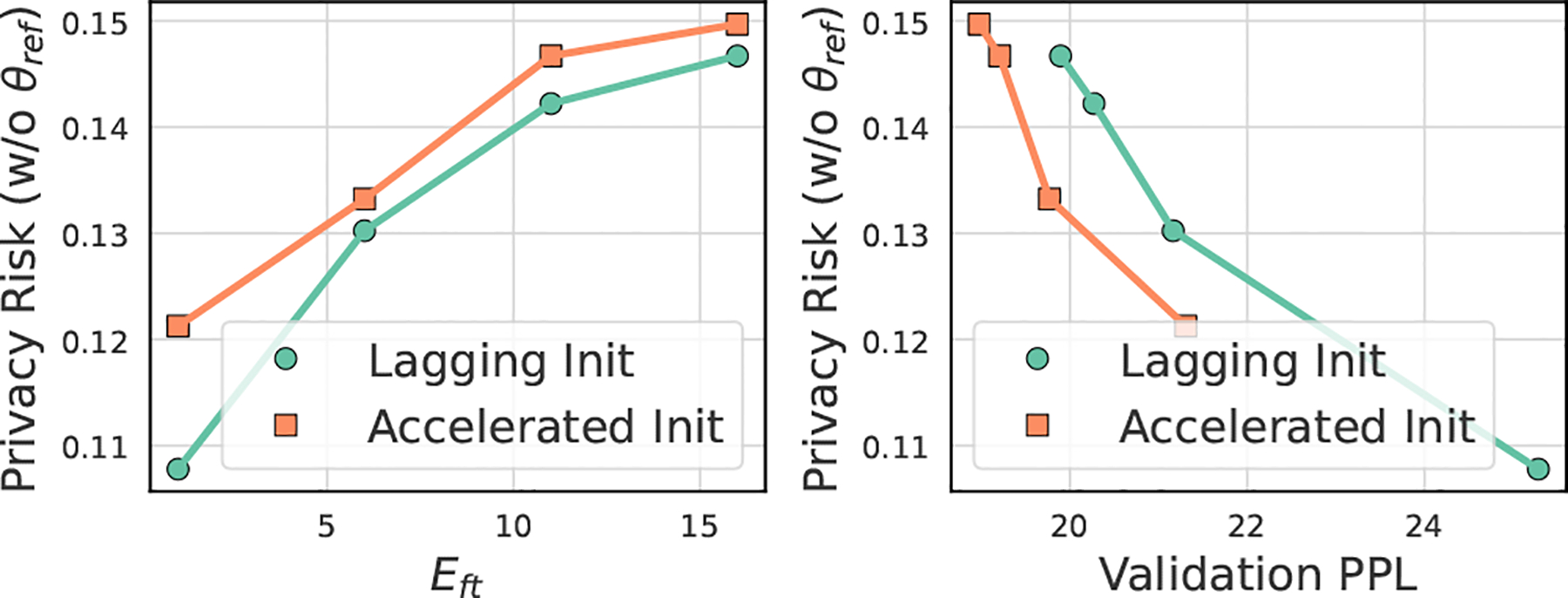
Privacy risk for different model initialization status. Each point indicates the fine-tuned checkpoint for the Enron dataset with Adapter-FT. We use TPR@0.1FPR as the proxy metric to measure the privacy risk of the model based on the scoring method in Equation (4). We fully-finetuned the benign GPT-2 model on the auxiliary dataset for $E_{\text{pre}}=1$ and $E_{\text{pre}}=5$ separately for *Lagging Init* and *Accelerated Init* with learning rate $\eta_{\text{pre}}=10^{-5}$ as model initialization.

Based on previous lessons on memorization [4, 34], it is intuitive that using a better trained model as initialization induces over-fitting on fine-tuning data, leading to higher privacy leakage via MIA or data extraction. However, if we consider two models that achieve the same performance after fine-tuning, but one spends more iterations and the other spends fewer iterations, the intuition turns into the opposite: initializing with a less trained model may have a higher privacy risk because it will take more iterations for the model to achieve the same desired performance and the model will have seen the data more times and the influence of a sample is greater. Both directions seem reasonable, we use the toy example in Figure 3 to show that the stopping criterion C is crucial for which intuition can lead to the success defined in Definition 2.1.

- **Case I.** In our default setting with the criterion $c_{\text{epoch}}$, fine-tuning stops within arbitrary fixed epochs known only to the victim. We expect a model initialization with higher memorization level leads to a higher privacy risk. The **left** figure in Figure 3 confirms this intuition, since the privacy risk of *Accelerated Init* given the same number of fine-tuning epochs is higher.
- **Case II**. We consider another case where performance based early-stopping is used to avoid overfitting, for example, the fine-tuning stops when the validation performance achieves a certain level. In the **right** figure of Figure 3, we can observe that the *Lagging Init* has a higher privacy risk given the same validation PPL. Our insight is that a lagging initialization pushes fine-tuners to train more iterations for achieving the same performance, implicitly increasing the number of duplicates for training samples, which has been shown as a cause of higher privacy risk [23].

By considering the stopping criterion when crafting the model initialization, our key intuition is to control the memorization stage for the model initialization on *Lagging* and *Accelerated* directions accordingly for achieving Definition 2.1.

### 3.2 Methodology for Crafting

Starting from the key intuition, we now introduce methodologies for controlling the two directions. The accelerating by warm-up (Section 3.2.1) and anti-freezing strategy (Section 3.2.2) are proposed for **Case I** while the lagging strategy (Section 3.2.3) is proposed for **Case II**.

#### 3.2.1 Accelerating by Warm-up (Case I).

With no knowledge of specific PEFT methods in ${𝒯}_{\text{ft}}$, we propose a basic method for accelerating the memorization stage in the fine-tuning data domain 𝒟 by fully fine-tuning on $D_{\text{aux}}$. Thus, for *selective* PEFTs such as Head-FT or Bitfit-FT, the starting point for these trainable parts is already optimized for the domain 𝒟, further tuning on these parameters can focus on learning the residuals or adjustments necessary to adapt the already domain-tuned representations of the base model to the nuances of $D_{\text{ft}}$.

For *additive* PEFTs such as Adapter [13] and *reparameterization-based* PEFTs such as LoRA [14], the inserted modules and low-rank matrices are usually randomly initialized by the victim. It will take some iterations for these randomized parts to fit and enter the memorization-only stage, but it is still faster than fine-tuning on $\theta_{\text{pre}}^{\text{benign}}$ that is pre-trained over the out-of-domain public data.

#### 3.2.2 Accelerating by Anti-freezing (Case I).

When the victim follows the guidance provided by 𝒜 on the choice of PEFT, 𝒜 can utilize this side information for pushing the released model initialization $\theta_{\text{pre}}$ to the memorization-only stage with a more aggressive acceleration.

In typical *addictive* and *selective* PEFT training, only the small and random inserted modules are trainable while keeping the rest pretrained parameters frozen. On the contrary, we freeze the inserted / reparameterized modules and tune the backbone in our crafting stage. The intuition is to make the released model equipped with a known PEFT module perfectly fit the data domain at the first step of ${𝒯}_{\text{ft}}$. Thus, the first fine-tuning step enters the memorization-only stage [34] and the privacy risk will increase rapidly.

It should be noted that there is still a small amount of randomness because the PEFT modules initialized in ${𝒯}_{\text{adv}}$ by the adversary are different from the one initialized in ${𝒯}_{\text{ft}}$ by the victim if the random seed is not fixed. Thus, we shift the seed in the two stages when performing the accelerated experiments for considering the influence of randomness. By our observation, changing the seed causes subtle differences and does not affect the effectiveness, which may be because the randomly initialized modules are drawn from a common distribution.

#### 3.2.3 Lagging by Weight Scaling (Case II).

In the opposite direction, for creating a lagging model initialization for privacy risk amplification in Case II, the intuitive idea is to make $\theta_{\text{pre}}$ perform worse or farther away from the data domain.

Ideally, learning a well-performed model is hard but hurting the utility is easy to achieve by simply spoiling the pre-trained parameters in $\theta_{\text{pre}}^{\text{benign}}$ with random noise, which does not even need the auxiliary knowledge $D_{\text{aux}}$. However, an even perturbation on a well generalized pre-trained model cannot specifically manipulate the memorization stage on the fine-tuning domain.

For better control of the memorization stage towards the fine-tuning domain, we propose scaling a portion of parameters in the warmed-up backbone with a scaling factor β, which can be seen as an approximation of dropout [42]. In each layer of a transformer-based backbone, there is a crucial component of multi-head self-attention (MHA). Given a sequence of l vectors $C\in R^{l\times d}$ and a query vector $q\in R^{d}$, the MHA output is:

(1) $$\text{Attn}\left(\text{Q},\;\text{K},\;\text{V}\right)=\text{softmax}\left(\frac{\text{Q}{\text{K}}^{⊤}}{\sqrt{d_{k}}}\right)V,$$

(2) $${\text{head}}_{i}=\text{Attn}\left(\text{q}{\text{W}}_{q}^{\left(i\right)},\text{C}{\text{W}}_{k}^{\left(i\right)},\text{C}{\text{W}}_{v}^{\left(i\right)}\right),$$

(3) $$\text{MHA}\left(\text{C},\text{q}\right)=\text{Concat}\left({\text{head}}_{1},\cdots ,{\text{head}}_{h}\right)W_{o},$$

where $W_{q}^{\left(i\right)},\;W_{k}^{\left(i\right)},\;W_{v}^{\left(i\right)}\in R^{d\times d_{h}}$ and $W_{o}\in R^{d\times d}$.

Thus, if we use β∈(0,1) to scale weights $W_{q},\;W_{k},\;W_{v}$, the magnitudes of the Q, K, and V vectors in Equation (1) will decrease by a factor of β. And the attention weights are more evenly distributed. Additionally, scaling down $W_{o}$ reduces the output magnitude and also hurts the expressiveness. Therefore, a pre-trained model after weight scaling will result in a worse initial performance compared to a benign model. We can apply the weight scaling strategy on the checkpoint after basic warm-up or after the accelerated strategy of anti-freezing for making the memorization degradation more specific to the domain 𝒟.

On the one hand, it makes the model run more iterations to achieve the required performance. On the other hand, the inserted small PEFT modules are encouraged to compensate for the reduced magnitude and expressiveness with a larger gradient magnitude.

#### 3.2.4 Rewinding for Stealthiness.

Since the victim might be suspicious of the crafting behavior, we propose to evade the abnormal values on proposed stealthiness metrics in Section 2.3.3. Rewinding [30] has been taken as a way to diagnose memorization in a neural network by replacing the weights of a single layer with an old version during training.

Our intuition for ensuring stealthiness is to find a controller for balancing the crafted version and a benign version. Thus, for a crafted model $\theta_{\text{pre}}^{adv}$, we rewind a layer to its old version in $\theta_{\text{pre}}^{\text{benign}}$. By controlling which layer and how many layers are rewound, we can trade off between stealthiness and attack effectiveness.

### 3.3 Inference Pipeline

#### 3.3.1 Membership Inference Pipeline.

In the inferring stage, we consider two standard membership scores for maximizing the adversary advantage in distinguishing the IN-world when z=x and OUT-world when z=⊥.

For the weakest adversary with no auxiliary dataset, loss value is a conventional signal for classifying samples as a member [50]:

(4) $$A_{\theta }(x)=I[ℒ(x;\theta )<\gamma ].$$

For an adversary with an auxiliary dataset or equally the predominant adversary 𝒜 in our case, we follow the state-of-the-art attacks [3, 34, 39, 48] and calibrate the membership score with a difficulty score, which can be estimated with an OUT-world reference model $\theta_{\text{ref}}$ trained with the auxiliary dataset. Thus, the signal for classification becomes:

(5) $$A_{\theta }\left(x\right)=I\left[ℒ\left(x;\theta \right)-ℒ\left(x;\theta_{\text{ref}}\right)<\gamma \right].$$

As previous works [33, 34], we threshold the above two signals by setting γ as the highest value of which the false positive rate over all samples would not exceed α for reporting the TPR with a given α FPR. We omit the discussion on estimating the difficulty score by a pool of reference samples [32] because loss-value and reference-model scores have already covered the lower and upper bound of empirical MIA performance. With the efficiency bottleneck on training multiple reference models, we limit the capability with only one reference model in all comparisons.

#### 3.3.2 Data Extraction Pipeline.

We perform the data extraction in the inferring stage based on a state-of-the-art pipeline [6] with two phases. In the generation phase, the adversary will query the target model to generate a large amount of text with or without a given prefix. In the membership inference phase, the adversary will sort the generated samples concerning Equation (4) or Equation (5) after deduplicating abnormally repeated samples.

## 4 Experiments

### 4.1 Experimental Setup

#### Datasets.

We run experiments on benchmark datasets from financial, email, and medical domains due to the confidential properties of the content, including Penn Treebank [31] (PTB), Enron [21] and Pubmed [9].

We split the original training dataset equally into three partitions as $D_{\text{ft}},\;D_{\text{aux}}$, and the non-member dataset $D_{\text{non}}$. Thus, we avoid a false sense of attack effectiveness from the potential data shift [16]. To control the strength of this adversarial knowledge, we vary the data size ratio between the auxiliary dataset and the fine-tuning dataset $r_{\text{aux}}=\left|D_{\text{aux}}\right|/\left|D_{\text{ft}}\right|$ and by default $r_{\text{aux}}=1$ as the other work [44]. For a fair comparison, we ensure same datasets are used in comparisons.

#### Models and Parameter-Efficient Fine-Tuning.

For the scalability to different backbone model sizes, we perform experiments on GPT-2 (12-layer, 117M), GPT-2-medium (24-layer, 345M), and GPT-2-large (36-layer, 774M) models. Except for fully fine-tuning (Full-FT), we extend our evaluation to two *selective* methods of Bitfit-FT and Head-FT, one *addictive* method of Adapter-FT in the output layer with a reduction factor as 16 and one *reparameterization*-*based* method of LoRA-FT with r=16.

We set a default learning rate η in Full-FT, Adapter-FT, LoRA-FT, Bitfit-FT, and Head-FT as $\left\{1e^{-5},1e^{-4},5e^{-4},5e^{-4},1e^{-4}\right\}$ with the linear scheduler in all baselines for a fair comparison. By default, we train the model with $E_{\text{ft}}=20$ on GPT-2, $E_{\text{ft}}=5$ for GPT-2-medium/large and stop without overfitting.

#### Baselines.

For the main goal of verifying if *PreCurious* enlarges the adversarial gain as we defined in Definition 2.1, we compare the privacy risk of $\theta_{\text{ft}}^{\text{adv}}$ and $\theta_{\text{ft}}^{\text{benign}}$.

For all fine-tuned models, we use results w/ $\theta_{\text{ref}}$ to show risks for 𝒜 who is the prominent adversary and the pretrained model publisher who has $D_{\text{aux}}$. Results w/o $\theta_{\text{ref}}$ reflect risks from the potential weaker adversary ${𝒜}_{w}$ that can be anyone who queries the model but has no $D_{\text{aux}}$. Thus, we could see the maximum secrets that can be inferred, as well as the attacking lower bound for the maximum coverage of potential adversaries.

As for $\theta_{\text{ref}}$, we use the model initialization as a default reference model, which is denoted as Base-Ref. To control influence from calibration, we use $\theta_{\text{ref}}$ trained over the same $D_{\text{aux}}$ for benign baseline, which is denoted as Full-Ref. By default, we evaluate baselines under **Case I** and discuss **Case II** in Section 4.2.6 for the early-stopping scenario.

#### Metrics.

We use the perplexity on validation dataset Val-PPL ↓ to measure the utility of the fine-tuned model. As shown in Section 2.3.3, we use $S_{\text{mia}}↓,\;S_{\text{mem}}↓$, and $S_{\text{gap}}↓$ with suffix token length as 10 to measure the stealthiness of the released model. For privacy budget, we follow the widely applied setting $\delta =n^{-1.1}$ for all $\epsilon^{1}$. For AUC ↑ and TPR@FPR α%↑ in MIA, we vary the FPR from 0.0001 to 0.1. For untargeted data extraction, we vary the sub-sequence length by L={2,5,10,40,50}. For $v_{\text{exp}}↑$ in targeted data extraction, we calculate the valid exposure threshold with the secret length of $L_{\text{secret}}=10$ characters.

### 4.2 Effectiveness on Membership Inference

In this section, we would like to measure the effectiveness of *PreCurious* on amplifying the membership inference risk with the following questions:

- **RQ1:** What is the extent of the advantage gained through *PreCurious* initialization compared to a benign one within the same iterations? (Section 4.2.1)
- **RQ2:** How does the choice of model initialization and reference model influence the adversarial advantage and interfere with each other? (Section 4.2.2)
- **RQ3:** Is the crafted backbone stealthy compared to the benign model? Which layer has more influence on stealthiness? (Section 4.2.3)
- **RQ4:** Which conventional defenses fail on mitigating privacy risk when applying *PreCurious*? (Section 4.2.4)
- **RQ5:** Does the risk amplification effect on MIA highly rely on the duplication between $D_{\text{ft}}$ and $D_{\text{aux}}$? (Section 4.2.5)
- **RQ6:** Can we break up the privacy-utility trade-off when early stopping is applied? (Section 4.2.6)

Denoting the learning rate, epochs in the **crafting** stage as $\eta_{\text{pre}},\;E_{\text{pre}}$, we now clarify variants of *PreCurious* as :

- **Basic** indicates the basic accelerating by warm-up (Section 3.2.1).
- **Accelerated** indicates accelerating by anti-freezing (Section 3.2.2).
- **Lagging** means releasing the model with inferior performance on the domain (Section 3.2.3). By default, it means the combination of anti-freezing backbone and weight scaling.
- **Stealthy** is the stealthier version for *Basic* by rewinding the head in the crafted backbone to the benign version (Section 3.2.4).

#### 4.2.1 Performance Comparison.

First, we summarize MIA performance between $\theta_{\text{ft}}^{\text{benign}}$ and $\theta_{\text{ft}}^{\text{adv}}$ in Table 1 from the lens of the prominent adversary 𝒜. Using a $\theta_{\text{ref}}$ trained over $D_{\text{aux}}$ significantly improves the attacking effectiveness on the benign baseline as shown in previous works [34, 39, 44]. Comparing with the state-of-the-art Full-Ref, we can see the adversary advantage is significantly amplified with a basic warm-up model initialization. This is because the *PreCurious*-Basic model initialization induces the fine-tuning process to start from a point close to the memorization-only stage [34] where membership inference risk increases rapidly and results in a higher privacy risk within given epochs.

Then, we evaluate the effectiveness of different backbones in Table 2. We use the same reference model for Basic and Full-Ref for fair comparison, and we set $E_{\text{ft}}=5$ on the two larger models to avoid showing results after overfitting. Comparing GPT-2 Medium with GPT-2 Large, under the same configurations, we can see that the Val-PPL and the MIA performance w/ or w/o $\theta_{\text{ref}}$ scales up with model size. Comparing *PreCurious*-Basic with Benign-Full-Ref, we can see that using a basic warm-up speeds up memorization and boosts the TPR@0.01%FPR for PTB dataset by ×18.84.

In addition, we observe the advantage introduced by model initialization in Table 3 by comparing Benign with Basic and the more aggressive Accelerated. We set $E_{\text{pre}}=1$ as a safe choice for the accelerated version on all datasets. There is a clear trend that the Val-PPL is decreasing and the privacy risk is increasing from Benign to Basic to Accelerated. The Accelerated is indeed a more aggressive strategy that pushes the starting point to memorization-only stage.

**RQ1-Response:** Whether with or without $\theta_{\text{ref}}$, the accelerated strategy of *PreCurious* enhances the MIA advantage across different PEFTs and model sizes within the given number of iterations.

#### 4.2.2 Ablation Study.

**Figure 4:**
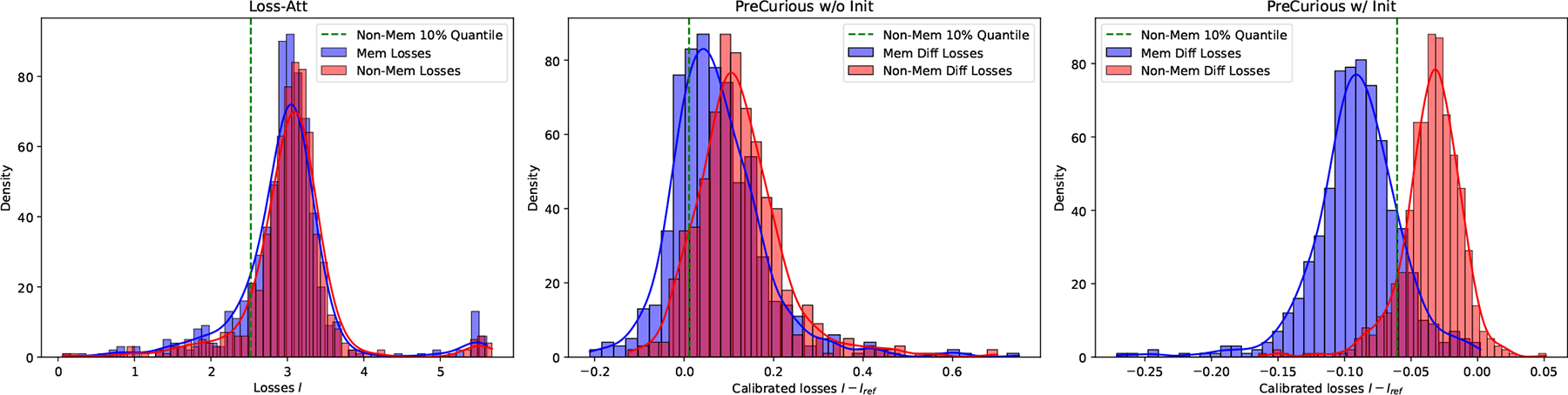
Ablation study of PreCurious on the crafted initialization and reference model with Enron and Adapter-FT GPT-2. Loss distributions for Benign initialization w/o $\theta_{\text{ref}}$, benign initialization w/ Full-Ref, and PreCurious initialization w/ Full-Ref.

**Figure 5:**
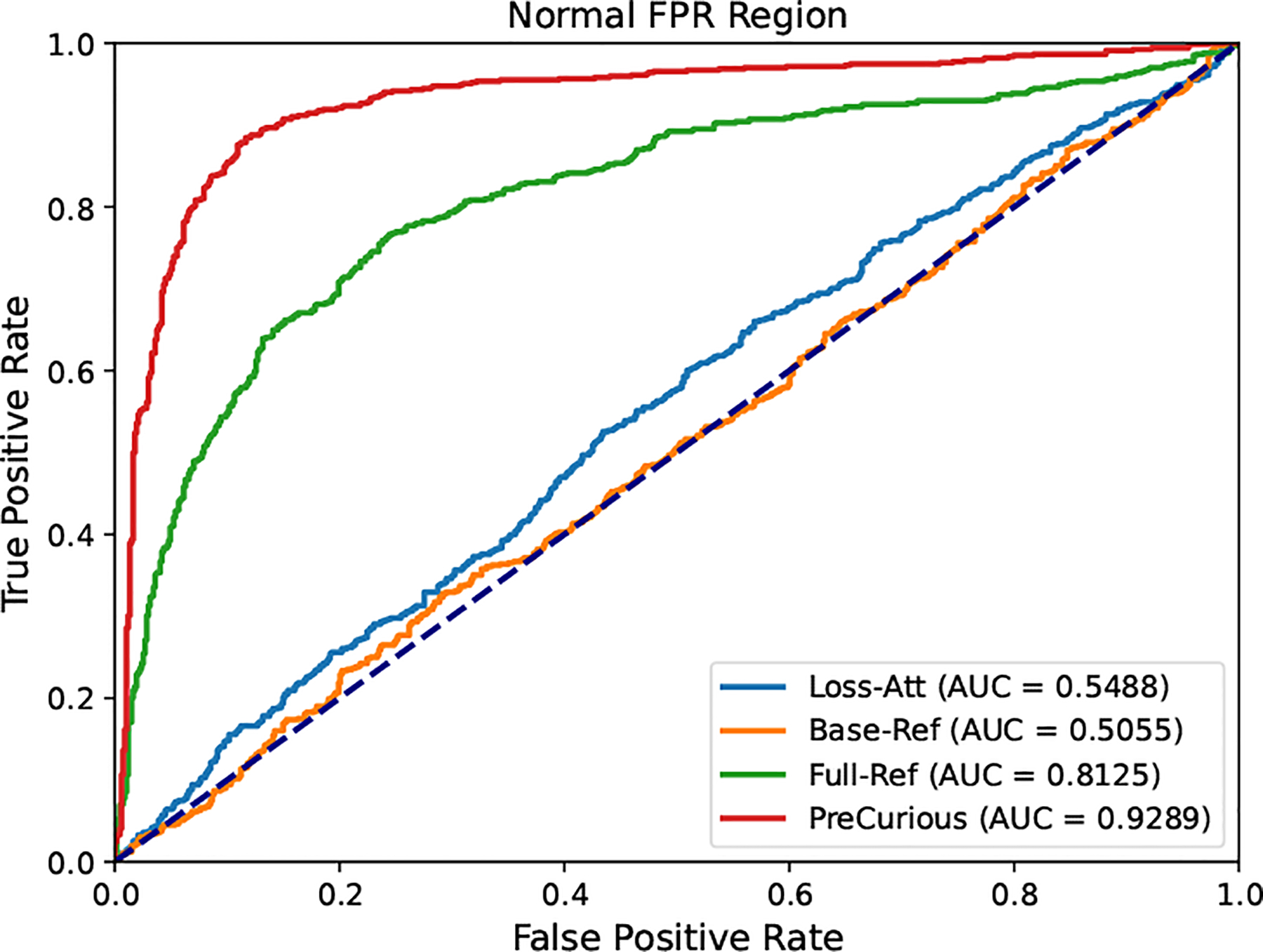
ROC-AUC curve for Enron on Adapter-FT GPT-2. Base-Full indicates calibrating with a benign model cannot even beat Loss-Att with the same benign initialization.

To show the independent advantage gained from the crafted initialization $\theta_{\text{pre}}^{\text{adv}}$ and the reference model $\theta_{\text{ref}}$, we perform an ablation study in Figure 4, in which we choose the best reference model for achieving the highest MIA AUC on Benign-Full-Ref baseline. First, the loss distribution shows the MIA signal distribution can be distinguished more significantly between members and non-members by adversarially crafting the initialization. Then, comparing the ROC curve of PreCurious with Benign-FullRef, we can see the small advantage w/o $\theta_{\text{ref}}$ in Table 3 is amplified after calibration. And we notice that the performance of calibration is highly sensitive to the choice of $\theta_{\text{ref}}$, as shown in Figure 5.

**Figure 6:**
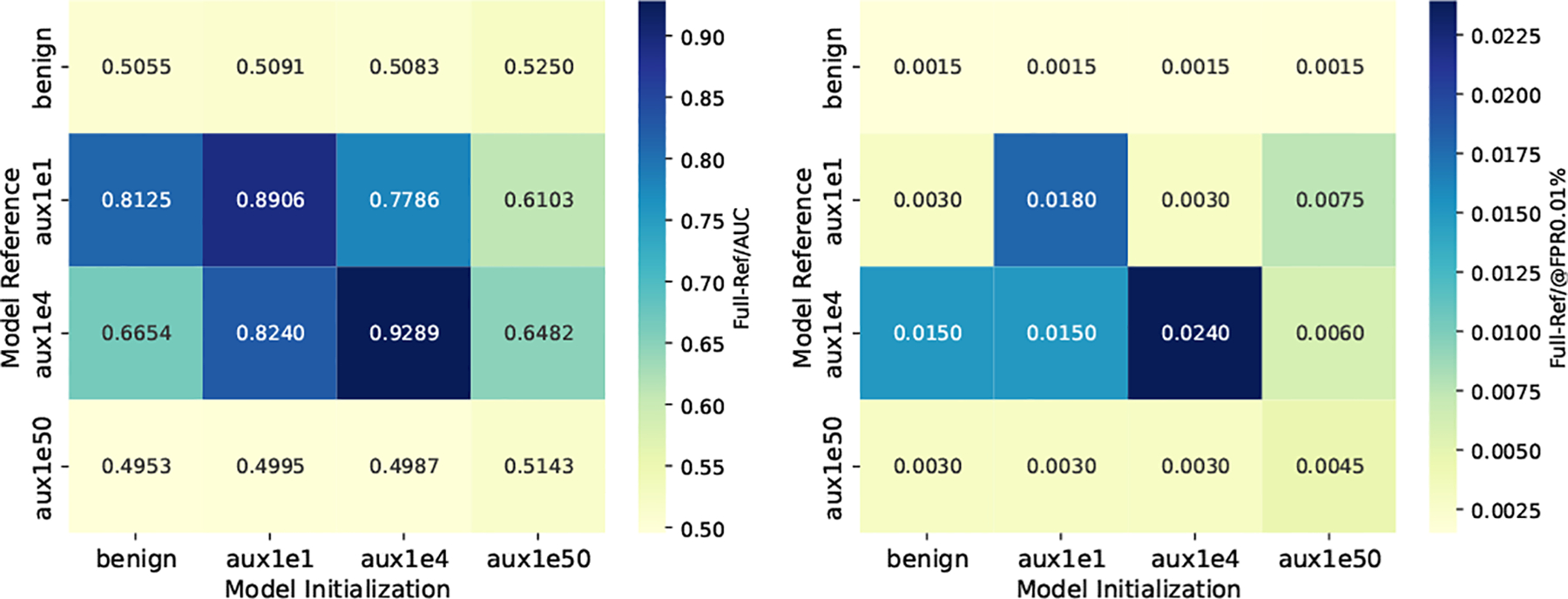
Influence of initialization and reference model choices on MIA success metrics (AdapterFT-Enron). aux1e1 (under-fit), aux1e4 (just-fit) and aux1e50 (over-fit) denotes checkpoints warmed up on $D_{\text{aux}}$ with Full-FT in the crafting stage of *PreCurious* to represent different overfitting levels on $D_{\text{aux}}$. We set a default $\eta_{\text{pre}}=10^{-4}$ for fully fine-tuning in ${𝒯}_{\text{pre}}$ to reduce the required $E_{\text{pre}}$ when simulating the overfitting status here.

Now we would like to discuss the best choice of $\theta_{\text{pre}}^{\text{adv}}$ and $\theta_{\text{ref}}$ for maximizing the MIA signal distinguishability, using PreCurious-Basic as an instance for the accelerated version. To understand how different choice of model initialization and reference model influence the adversarial advantage, we combine different warming-up checkpoints as $\theta_{\text{ref}}$ and $\theta_{\text{pre}}^{\text{adv}}$ in Figure 6. First, we find a consistent rule that the best $\theta_{\text{pre}}^{\text{adv}}$ and $\theta_{\text{ref}}$ combination for achieving the maximum advantage across different MIA metrics, datasets, and PEFTs is aux1e4-aux1e4. Also, there is a clear trend that diagonal combinations yield higher risk, indicating the best $\theta_{\text{ref}}$ is $\theta_{\text{pre}}^{\text{adv}}$ or the one that has a slightly better performance to $\theta_{\text{pre}}^{\text{adv}}$. Since the attack effectiveness of referenced model-based MIA is significantly influenced by the choice on $\theta_{\text{ref}}$, our finding solves the challenge by providing a simple rule of choosing $\theta_{\text{ref}}$.

**RQ2-Response: 𝒜** is suggested to use the just-fit model as $\theta_{\text{ref}}$ and $\theta_{\text{pre}}^{\text{adv}}$ in accelerated *PreCurious*.

#### 4.2.3 Stealthiness.

**Figure 7:**
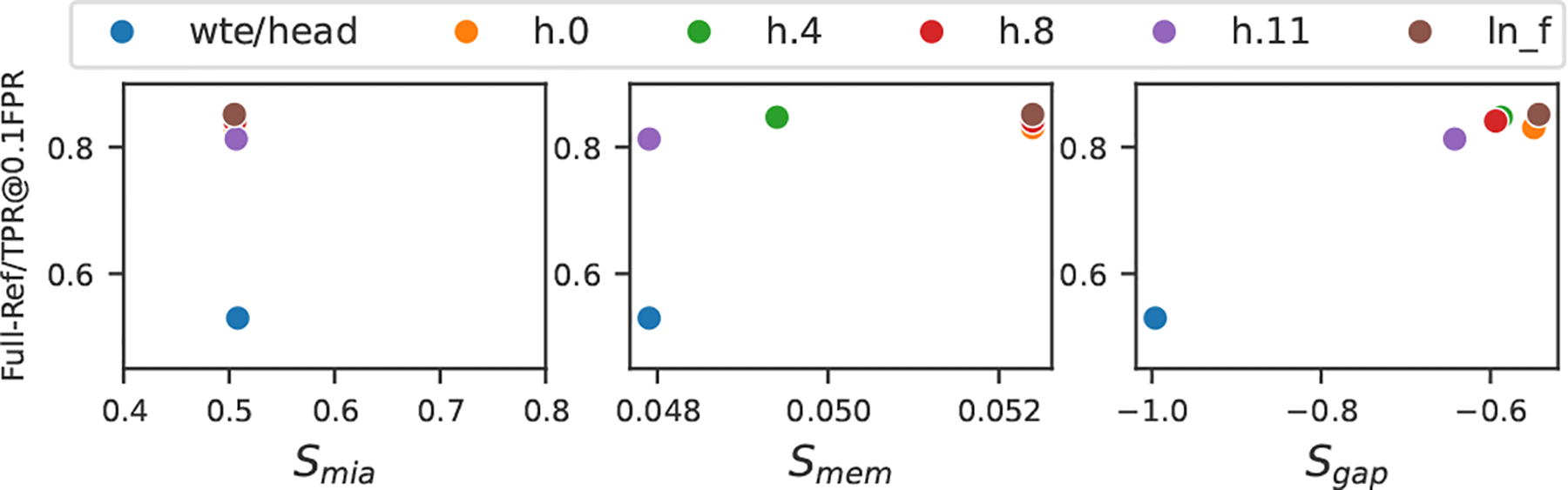
Stealthiness-Risk trade-off via rewinding layers on Enron dataset with Adapter-FT.

Now we suppose the victim doubts the motivation of $\theta_{\text{pre}}^{\text{adv}}$ and the victim can query the benign $\theta_{\text{benign}}$ for auditing. Thus, we compare the stealthiness metrics across benign backbone and *PreCurious* backbones in Table 4. First, the proposed stealthiness metrics are possible to raise suspicion for $\theta_{\text{pre}}^{\text{adv}}$ if the victim is sensitive to the subtle differences. $S_{\text{mem}}$ gives a more consistent detection compared to $S_{\text{mia}}$ or $S_{\text{gap}}$. Second, *Stealthy* is effective in enhancing the stealthiness of *Basic. Accelerated* is also stealthier than the *Basic* because auditing is performed on the backbone instead of composing with inserted modules. But as shown in Table 1, *Stealthy* sacrifices the attack effectiveness with the slight improvement on stealthiness. Third, *Lagging* has $S_{\text{mem}}=0$ and may successfully evade with $S_{\text{mia}}\approx 0.5$ and low $S_{\text{gap}}$, except for $S_{\text{gap}}$ on Enron. The high $S_{\text{gap}}$ results from the randomness of the poor initial utility. Performing layer-wise rewinding in Figure 7, we observe that rewinding the last block can achieve the best stealthiness-risk trade-off.

**RQ3-Response:** *PreCurious* increases stealthiness metrics very subtly and 𝒜 can rewind the last block to further enhance the stealthiness.

#### 4.2.4 Effectiveness under Defense.

Under the representative defense strategy of weight decay, we show in Table 5 that *PreCurious* is robust on privacy risk amplification even with a high coefficient that exceeds the typical selection.

Under the strict defense of DP fine-tuning [24, 51], we show in Table 6 that *PreCurious* model increases the AUC compared to the Benign model but has a smaller TPR@0.01FPR and better utility due to the warming-up. The overall risk compared to non-DP fine-tuning in Table 1 is significantly mitigated by DP, supported by more results w.r.t. various budgets in the Appendix Table 8.

**Figure 8:**
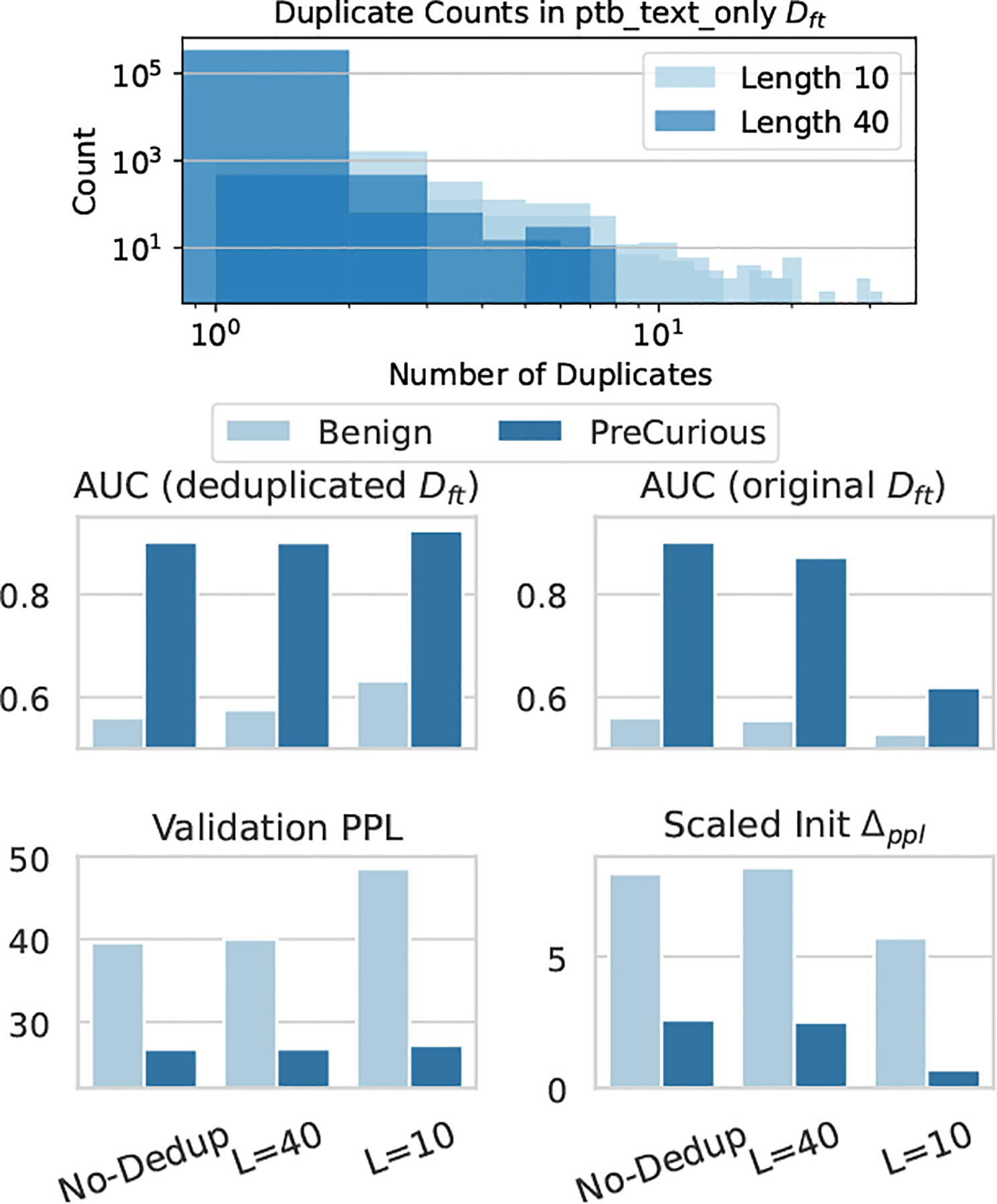
Duplication statistics and MIA effectiveness with Full-Ref under deduplication defense on $D_{\text{ft}}$ for PTB. The stealthiness metric $S_{\text{gap}}=\Delta_{\text{ppl}}$ is linearly scaled for clear visualization. We randomly subsample non-membership samples for keeping the same size as the deduplicated $D_{\text{ft}}$ in MIA evaluation.

In Figure 8, we evaluate the MIA effectivenss of Benign and PreCurious under deduplication defense [20, 23]. As shown in the duplicate statistics at the top, a sub-sequence in $D_{\text{ft}}$ may appear multiple times and make it easier to memorize [20]. Deduplication can be instantiated with suffix array-based algorithm [23] for finding and mitigating repeated sub-sequences in $D_{\text{ft}}$.

By deduplicating repeated sub-sequence of length L={10,40} in $D_{\text{ft}}$, we find a consistent trend that *PreCurious* still causes a higher MIA risk than Benign initialization. Taking original $D_{\text{ft}}$ as members, heavier deduplication leads to less privacy risk. But we note that *PreCurious* with a heavy deduplication such as L=10 still causes more privacy leakage than Benign baseline without deduplication. Also, deduplication helps 𝒜 to be more stealthier and results in a higher perplexity (worse utility-privacy trade-off), because the auxiliary dataset is not deduplicated. When taking samples in deduplicated $D_{\text{ft}}$ as members, the MIA risk is increasing for a heavier deduplication due to a larger distribution shift. This is also because the data size used for fine-tuning is diminished and the deduplication essentially induces training samples to become outliers and more vulnerable to be inferred [44]. The ideal case where attackers can approximate deduplicated texts in MIA inference can be seen as a corner case for deduplication defense to fail.

**RQ4-Response:** *PreCurious* still effectively amplifies the privacy risk under defenses and is even stealthier under deduplication.

#### 4.2.5 Duplicates Investigation.

In previous experiments, we use a randomly split dataset as $D_{\text{aux}}$ for launching *PreCurious*. However, $D_{\text{aux}}$ may have partially overlapped sub-sequence as in $D_{\text{ft}}$, which might be the reason for a successful privacy risk amplification. To understand whether the risk amplification effect is highly dependent on the duplication between the two datasets $D_{\text{ft}}$ and $D_{\text{aux}}$, we control the overlapping level of $D_{\text{aux}}$ with cross-deduplication:

- For $D_{\text{aux}}^{\text{dedup}}$, we **drop** all L-length sub-sequences that overlaps with $D_{\text{ft}}$ on the default $D_{\text{aux}}$.
- For $D_{\text{aux}}^{\text{dup}}$, we find all cross-duplicated L-length sub-sequences and **keep** them to construct it.

By varying over different L={2,5,10,40,60}, we get $D_{\text{aux}}^{\text{dup}}$ and $D_{\text{aux}}^{\text{dedup}}$ with various auxiliary dataset sizes. It should be noted that this experiment is designed for analysis instead of a “real” attack as we are manipulating the adversary capability with $D_{\text{ft}}$.

**Figure 9:**
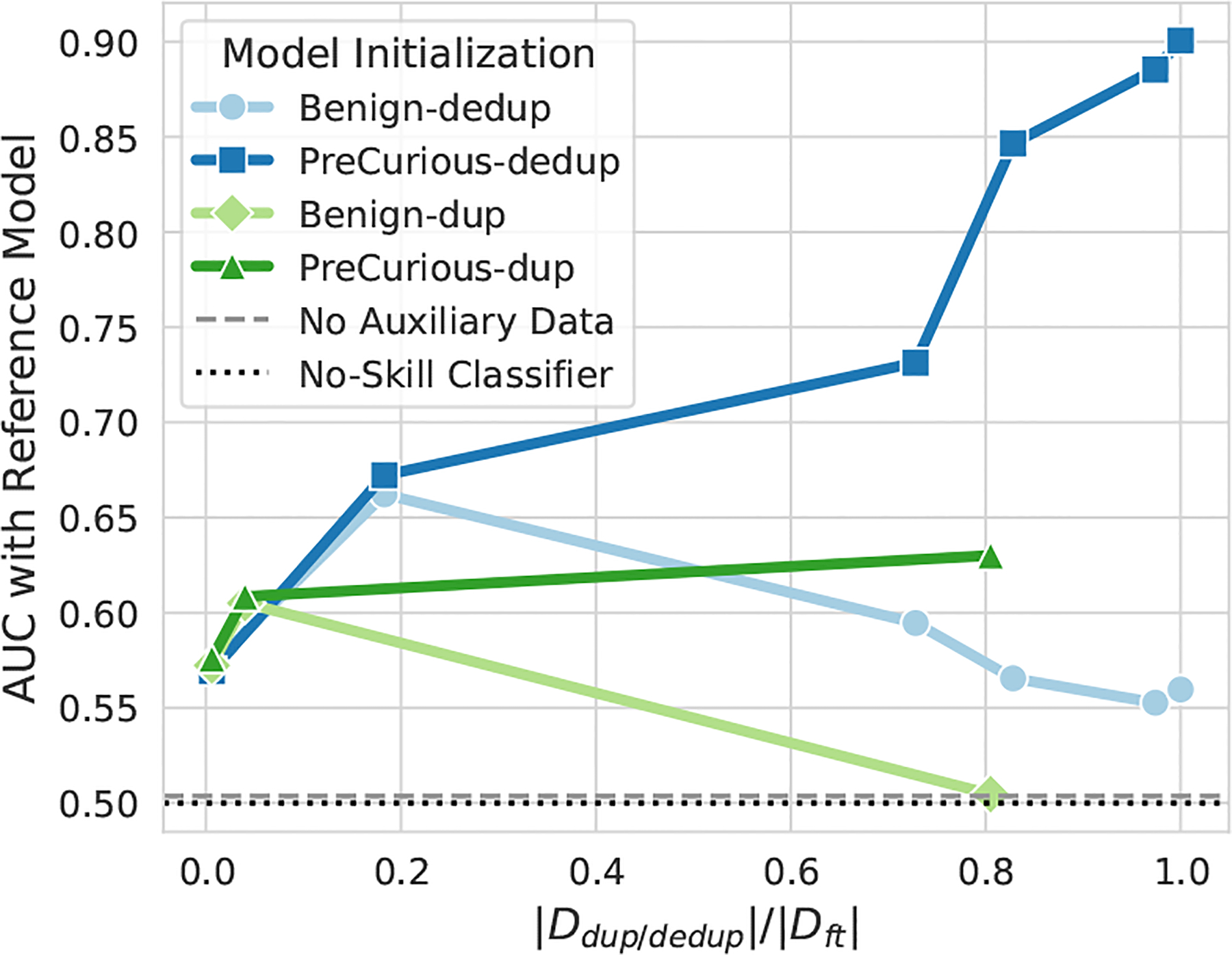
MIA effectiveness on PTB dataset with $D_{\text{aux}}^{\text{dedup}}$ and $D_{\text{aux}}^{\text{dup}}$ as auxiliary data for training $\theta_{\text{ref}}$ in Benign or $\theta_{\text{pre/ref}}$ in PreCurious, and $\left|D_{\text{dedup}/\text{dup}}\right|/\left|D_{\text{ft}}\right|=1$ denotes the default $D_{\text{aux}}$ w/o deduplication.

As shown in Figure 9, we control the duplication level by increasing L for $D_{\text{aux}}^{\text{dedup}}$ and decreasing L for $D_{\text{aux}}^{\text{dup}}$ from left to right. We can observe that using the auxiliary knowledge with $D_{\text{aux}}^{\text{dedup}}$ has superior attack performance than $D_{\text{aux}}^{\text{dup}}$, which indicates that the privacy risk amplification of *PreCurious* does not solely rely on the cross-duplicated parts between $D_{\text{aux}}$ and $D_{\text{ft}}$. Then, we observe a clear trend for all datasets that the adversarial advantage of *PreCurious* with auxiliary knowledge $D_{\text{aux}}^{\text{dedup}}$ increases with a moderate level of cross-deduplication, with a similar trend shown for Benign baseline with $\theta_{\text{ref}}$. In addition, by only using the duplicated parts, which are typically the very common sub-sequences in the domain 𝒟, even the adversarial gain from $\theta_{\text{ref}}$ is poor, warming up with a batch of common fragments also helps to amplify the MIA risk, which weakens the required assumption on $D_{\text{aux}}$.

**RQ5-Response:** *PreCurious* does not heavily rely on the duplicates between $D_{\text{ft}}$ and $D_{\text{aux}}$.

#### 4.2.6 Breaking-up the trade-off.

As shown in Figure 11, we can use lagging *PreCurious* to break up the privacy-utility trade-off and amplify the risk for Case II. We compare all baselines with loss signals to avoid the influence of $\theta_{\text{ref}}$. We can observe that *PreCurious*-Lagging w/ $D_{\text{aux}}$ is possible to amplify the risk. But only weight scaling on a benign backbone is not as effective as scaling with the same level on a warmed-up model to distinguish the loss signal distribution at the end, validating the effectiveness of anti-freezing.

It is seen that *PreCurious*-Accelerated shows a consistent tendency to amplify risk given fixed epochs $E_{\text{ft}}$. While *PreCurious*-Lagging is robust in breaking up the privacy-utility trade-off, resulting in either poor model performance or high privacy risk, which validates our key intuition of increasing risk by increasing the required iterations to achieve the same utility. One different observation is that applying a lagging initialization for LoRA-FT does not show the same sign to amplify risk given a fixed epoch as expected. In addition, we find weight scaling with β=0.1 on attn.c_attn.weight is effective while the effective choice for Adapter-FT is attn.c_proj.weight, which are exactly where PEFT modules are applied, indicating the importance of fine-tuning side-information for the lagging strategy.

**Figure 10:**
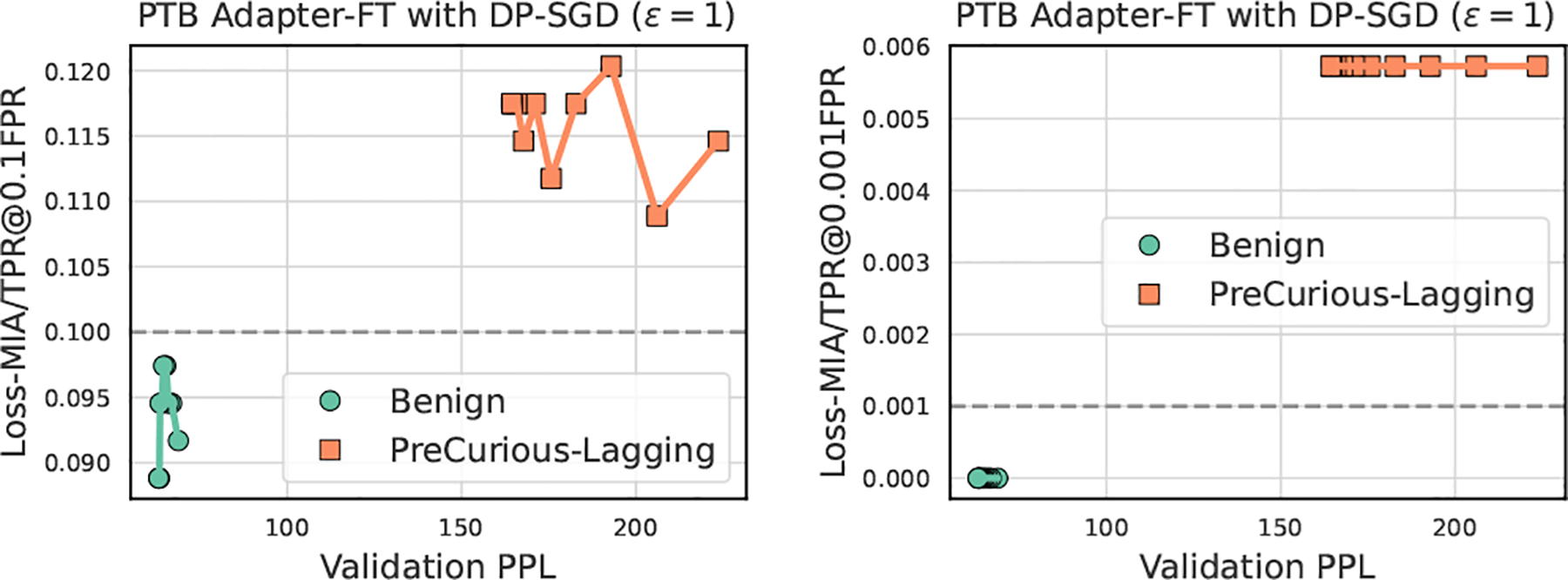
Breaking up privacy-utility trade-off under DP.

**Figure 11:**
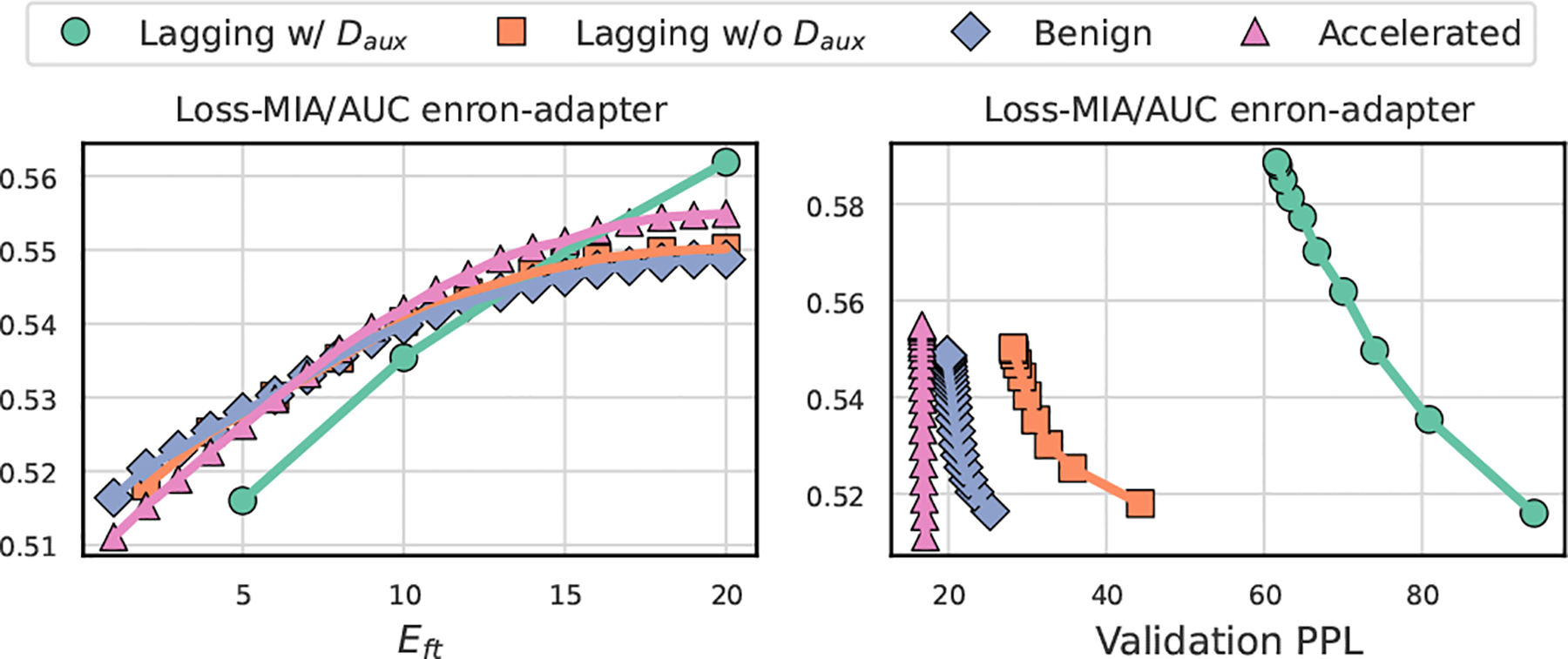
MIA effectiveness for Enron and PTB datasets with Adapter-FT. The baseline of Lagging w/ $D_{\text{aux}}$ indicates anti-freezing on $D_{\text{aux}}$ and then applying weight scaling with β=0.1. We use different seeds when randomly initializing adapter module parameters for ${𝒯}_{\text{pre}}\;\text{and}\;𝒯$. Lagging w/o $D_{\text{aux}}$ performs the weight scaling directly on the benign $\theta_{\text{benign}}$.

In addition, we address the privacy-utility trade-off issue in Table 6 with the lagging strategy as shown in Figure 10. Even when the worst-case privacy is bounded by a strict DP, we show that ϵ=1 is still not a perfect protection. This success is due to more iterations for achieving the same utility, and also because the larger gradient norm derived from *PreCurious*-lagging fully exploits the per-sample sensitivity to reflect the influence of each sample.

**RQ6-Response: 𝒜** is suggested to apply Lagging-*PreCurious* for breaking-up utility-privacy trade-off when early stopping is applied.

### 4.3 Effectiveness on Data Extraction

Now we evaluate the effectiveness of *PreCurious* on data extraction. As previous work [5, 20, 23] conclude, less duplicated secrets are more challenging to be extracted, thus we raise questions:

- **RQ7:** Are less deduplicated training samples safe with DP training and constraint of limited query times? (Section 4.3.1)
- **RQ8:** How bad is *PreCuious* when maximizing the auxiliary knowledge? (Section 4.3.2)

#### 4.3.1 Untargeted Extraction.

For **RQ7**, we focus on the effectiveness of samples of less duplication in $D_{\text{ft}}$ and assume the victim applies DP fine-tuning with ϵ=0.05 and the target can only query for limited 1,000 generations. We perform the untargeted extraction in Section 3.3.2 for both Benign and *PreCurious* by: 1) generating samples with a maximum length of 512 via length 200-length prefixes, and 2) deduplicating and ranking by MIA signals in Equation (5) to filter 100 samples. The prefixes are constructed by using the top frequent phrases shown in $D_{\text{aux}}$ as we suppose the short but common parts can be transferred to $D_{\text{ft}}$.

**Figure 12:**
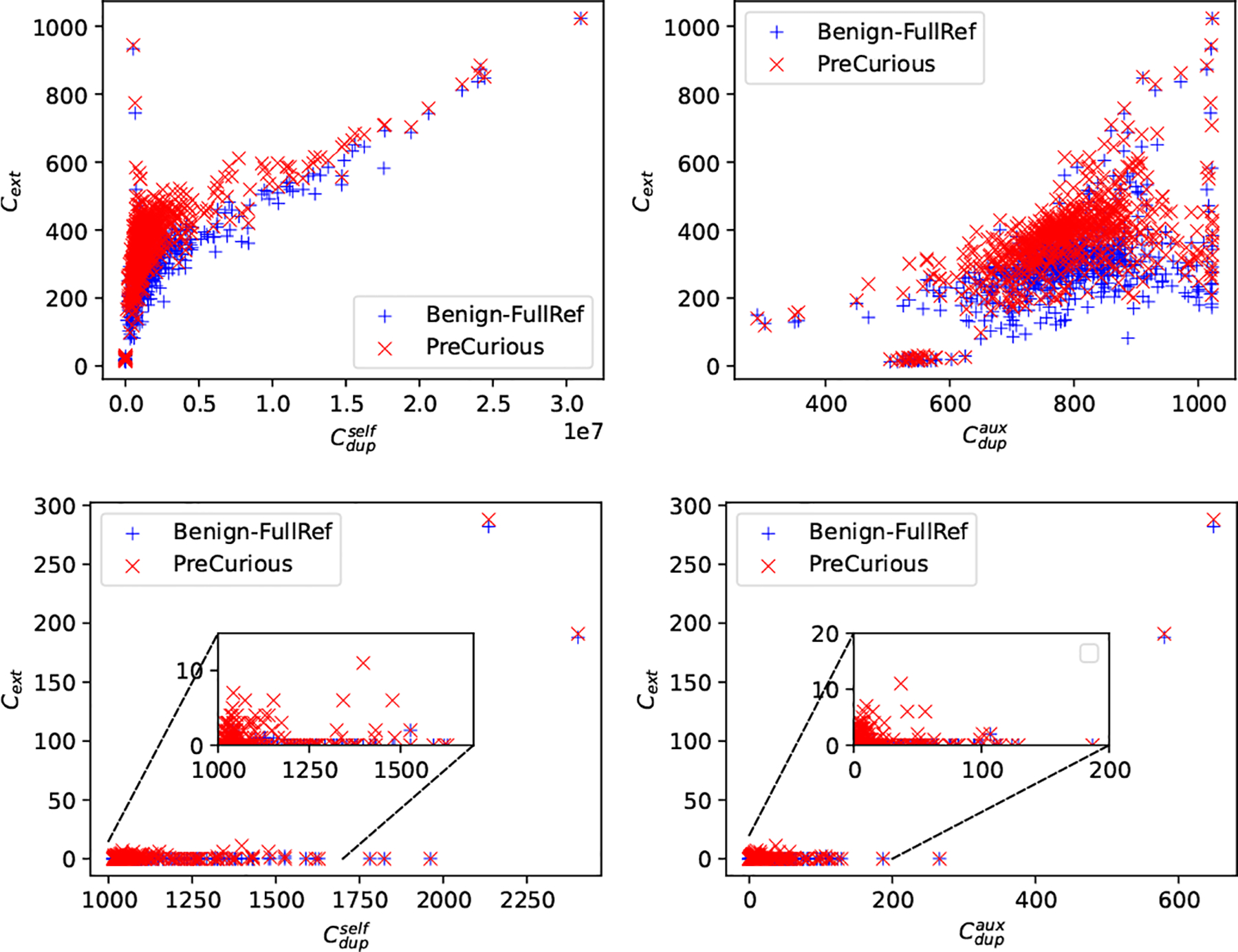
Untargeted Data Extraction for Adapter-FT model with $L_{\text{sub}}=2$ for for Enron (top) and $L_{\text{sub}}=10$ PTB (bottom).

In Figure 12, we use the $C_{\text{ext}}$ to denote the extraction level for **each sample** in $D_{\text{ft}}$, which counts the total times of its sub-sequences shown in all generated outputs. The averaged performance measure by $p_{\text{ext}}$ is shown in Appendix Table 9. $C_{\text{dup}}^{\text{self}}$ and $C_{\text{dup}}^{\text{aux}}$ indicate the total times of its sub-sequences shown in $D_{\text{ft}}$ and $D_{\text{aux}}$, respectively. In Figure 12, there is a clear trend that $C_{\text{ext}}$ increases with larger $D_{\text{dup}}^{\text{self}}$ and $D_{\text{dup}}^{\text{aux}}$, thus extracting less duplicates are indeed more challenging. But *PreCurious* can significantly improve the success on less duplicated samples, even under strict privacy defense given limited query times.

**RQ7-Response:** *PreCurious* can still increase leakage of fewer-duplicated secrets even with DP fine-tuning.

#### 4.3.2 Targeted Extraction.

**Figure 13:**
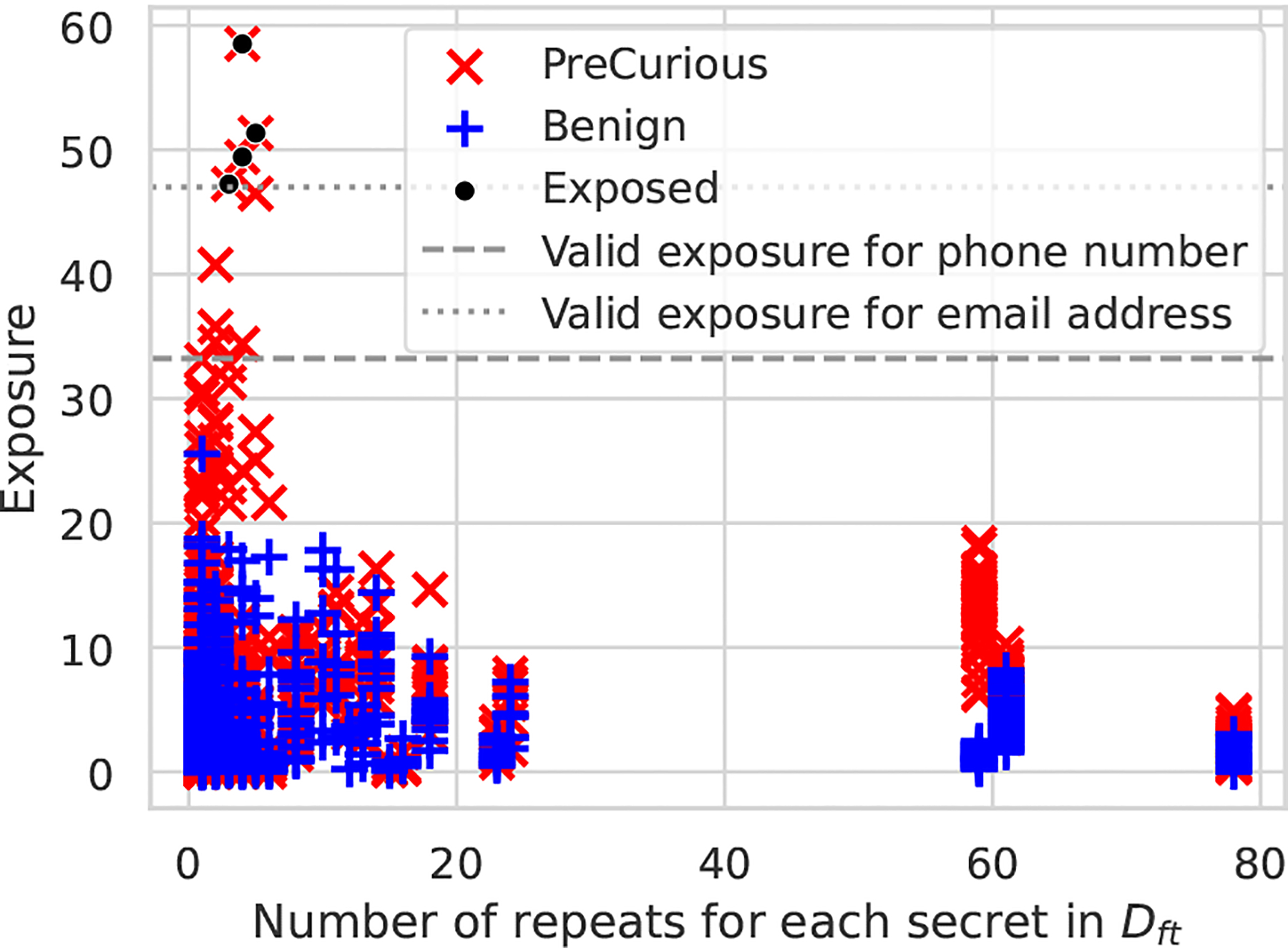
Targeted data extraction on Enron with Adapter-FT and ϵ=0.05 for DP-SGD. No secret’s exposure is above the valid threshold for fine-tuned benign model under DP.

To investigate the threat when 𝒜 in *PreCurious*, we design the targeted extraction with the Enron dataset and take the phone number and email addresses as our targeted secrets. For maximizing the auxiliary knowledge, we take a masked version of $D_{\text{ft}}$ as the $D_{\text{aux}}$, which is bold but possible because releasing de-identified text data is taken as a common practice [19]. After that, we apply *PreCurious*-Basic and evaluate the exposure on our targeted secretes for both $\theta_{\text{ft}}^{\text{adv}}$ and $\theta_{\text{ft}}^{\text{benign}}$. Following previous works [5, 34], we use the skew-normal distribution [36] to model the perplexity distribution of secrets for efficiently approximating the exposure. The precise exposure is upper-bounded by ${\text{log}}_{2}\;|ℛ|$ when the target secret ranks the first among the whole set of possible secrets ℛ. Thereby, the threshold ${\text{log}}_{2}\;|ℛ|$ on the approximated exposure discriminates the case where a secret is only marginally the most likely or the case a secret is beyond the most likely. A secret is only reliably extracted from the model with an exposure above the threshold [5]. More specifically, we take secret as 10 digits in phone numbers and 10 English characters in email, thus derive ${\text{log}}_{2}\left(10^{10}\right)\approx 33$ and ${\text{log}}_{2}\left(26^{10}\right)\approx 47$ as the valid exposure threshold. We can draw the following conclusion from Figure 13.

**RQ8-Response**: *PreCurious* can use sanitization text to expose originally safe secrets even when scrubbing is perfect.

## 5 Related Work

We discuss the most related attacks and privacy risk amplification.

### Membership Inference Attack.

MIA in machine learning context [41, 48] aims to predict whether a given sample is involved in training. Considering the inefficiency of LLM training, we focus on threshold-based MIA as it is more practical than attack-model-based MIA [8, 25, 35, 41]. The key idea of threshold-based MIA is formalizing a hypothesis test with the posterior distribution assumptions about the model parameters [3, 27, 48], by observing the signals from loss value [50] or the loss calibrated by other models or samples [3, 28, 32, 34, 39, 45]. Our evaluations integrate both conventional loss signal [50] and the state-of-the-art reference-model calibrated signal [3, 34, 48] without retraining or multiple queries for each sample for a practical adversarial capability assumption.

### Data Extraction.

Instead of extracting artificial canaries [5], a previous work [6] formulates the paradigm of extracting verbatim subsequence from the pre-training dataset of GPT-2 by filtering and ranking generated samples. We evaluate the verbatim extraction on real secrets under this paradigm.

### Privacy Risk Amplification.

The key idea of privacy risk amplification is to manipulate model or data integrity for more privacy leakage, as in representative works listed in Table 7. Prior works [7, 29, 44] investigate the privacy risk amplification via data poisoning, which requires the control of the training dataset. Recent work [43] attempts to enlarge the property inference effect by manipulating the pre-trained encoder for image classification. Our attack does not require control over the target training dataset and aims to plant a privacy backdoor in pre-trained model for amplifying general privacy risks in LLMs. Concurrent works [11, 47] also introduce privacy backdoors for pre-trained models, but [11] is not comparable to ours as they focus on classification task and mainly assume stronger capabilities of white-box and architecture modification. The other attack [47] is close to our basic version. Our advanced strategies further consider random PEFT initialization and early-stopping performed by the victim.

## 6 Discussion

### Countermeasures.

We now discuss the countermeasures to PreCurious for the wide range of users and regularization designers.

#### Be careful to download models from unknown sources.

The amplified risk from *PreCurious* justifies the importance of model integrity in pre-training and fine-tuning pipeline. Therefore, we recommend that fine-tuners download pre-trained models from trusted sources rather than from anonymous users on open-source platforms. Users should check the download link and be aware when automatic library management tools upgrade to higher version packages.

#### Be careful when following fine-tuning instructions.

With the rapid development of language models, users with different backgrounds can get started on building their models easily by following tutorials from the community. However, the success of *PreCurious* reveals additional side information that can be exploited by the adversary to infer private information. Users should not rely heavily on common settings shared in a tutorial, but instead be aware of the training dynamics in fine-tuning (e.g., epochs, stopping criteria, PEFT choices), even as the validation loss continues to decrease.

#### Be careful on auditing risks even under defense.

*PreCurious* demonstrates that regularization defense, DP fine-tuning, and deduplication are not perfect. For example, DP even with a strict budget cannot lead to a random guess attack under *PreCurious*; deduplication fails when attackers can approximate the deduplicated text in MIA, or when *PreCurious-lagging* implicitly increases the number of repetitions for all samples. Thus, we suggest that users remain vigilant and audit the privacy dynamics during fine-tuning closely [3, 5, 34] even when reasonable defenses are applied.

#### Be careful to share sanitized text by masking PII.

*PreCurious* demonstrates the feasibility of increasing the risk of secret exposure by using a public sanitized dataset to improve the auxiliary knowledge. Thus, we claim that unless we can ensure that sensitive information is removed for each future training, it is not safe to publish sanitized datasets, even if the sensitive secrets are masked or replaced, which is important when researchers in high-stakes domains publish benchmark datasets.

### Implications for future works.

A recent work [49] investigates the influence of model initialization on the worst-case privacy risk scales with the gradient difference on neighboring datasets and the iterations. *PreCuious* fills the gap between the theoretical discussion on model initialization from scratch and the practical use of pre-trained LMs and PEFT technique from an average case perspective. It is interesting for future work to improve the theoretical understanding of worst-case privacy when applying model efficiency techniques, as well as to exploit other side information to explore potential vulnerabilities for evaluating existing defenses.

From *PreCurious*, we note that memorization-based privacy backdoors on either accelerating or lagging direction should be coupled with the stopping criteria to derive the final risk amplification effect. Since there is no privacy attack considered to improve risks when victims perform early stopping, we bring new perspectives for future attacks and defenses under this realistic scenario. In addition, *PreCurious* reveals the vulnerability and identifies corner cases of existing defenses, providing a critical call for stronger defenses.

## 7 Conclusion

In this paper, we introduced *PreCurious*, a novel privacy risk amplification framework that increases the privacy risk of fine-tuning dataset by manipulating the pre-trained model’s memorization level and releasing a crafted model, showing the importance of model integrity from the privacy lens. We are among the first to investigate privacy backdoors, throughly exploring cases of PEFT and early-stopping by leveraging the side information in fine-tuning guideline. Our findings show that *PreCurious* breaks up the privacy-invulnerability property for PEFT, and common-sense defenses are possible to be subverted. Our work takes the step to understand the interplay between model memorization, efficiency and privacy risks, while also raises an interesting perspective to break up privacy-utility trade-off. This research is a critical call to action, urging the community to improve safeguards and reevaluate the security protocols around the use of pre-trained models, particularly those sourced from unverified platforms.

## Figures and Tables

**Table 1:** Membership inference evaluation on GPT-2 with various PEFTs $\left(E_{\text{ft}}=20,E_{\text{Pre}}=4\right)$. Loss-Att indicates loss-value based MIA in Equation (4) and Full-Ref indicates reference-model-based MIA in Equation (5). PreCurious shows amplified risk on all datasets, all PEFT methods in all MIA success metrics, while slightly increases the model performance measured by Val-PPL. PreCurious-Stealthy has an inferior attack performance than Basic but still amplifies risks compared to benign models.

Dataset			Enron				PubMed				PTB		
Adapter-FT		Val-PPL	AUC	@FPR1%	@FPR0.01%	Val-PPL	AUC	@FPR1%	@FPR0.01%	Val-PPL	AUC	@FPR1%	@FPR0.01%
PreCurious	Basic	17.19	92.89%	16.17%	2.40%	15.93	99.59%	92.34%	68.33%	23.16	99.79%	96.85%	92.84%
	Stealthy	17.86	82.42%	7.63%	1.80%	18.78	60.74%	2.66%	0.57%	25.37	93.00%	46.70%	14.90%
Benign	Loss-Att	19.84	55.00%	1.05%	0.00%	18.71	56.04%	1.47%	0.00%	30.43	56.97%	2.58%	2.29%
	Full-Ref	19.84	81.24%	8.53%	0.30%	18.71	75.25%	11.46%	0.52%	30.43	70.11%	16.62%	2.58%
Bitfit-FT		Val-PPL	AUC	@FPR1%	@FPR0.01%	Val-PPL	AUC	@FPR1%	@FPR0.01%	Val-PPL	AUC	@FPR1%	@FPR0.01%
PreCurious	Basic	17.33	76.20%	3.89%	0.75%	16.00	76.01%	6.70%	1.62%	23.18	94.90%	50.72%	40.40%
	Stealthy	18.77	59.06%	3.89%	0.45%	17.00	61.21%	3.80%	0.19%	25.99	71.24%	5.16%	1.72%
Benign	Loss-Att	22.07	52.55%	1.20%	0.00%	21.57	51.51%	1.19%	0.00%	35.74	52.14%	2.29%	2.01%
	Full-Ref	22.07	58.06%	4.64%	0.15%	21.57	55.08%	2.04%	0.00%	35.74	65.14%	6.02%	0.86%
LoRA-FT		Val-PPL	AUC	@FPR1%	@FPR0.01%	Val-PPL	AUC	@FPR1%	@FPR0.01%	Val-PPL	AUC	@FPR1%	@FPR0.01%
PreCurious	Basic	17.06	93.76%	17.37%	1.95%	16.83	94.12%	52.73%	22.35%	23.06	99.94%	97.99%	93.98%
	Stealthy	17.97	81.38%	8.83%	2.10%	15.94	99.72%	93.87%	69.42%	25.91	91.48%	36.39%	17.48%
Benign	Loss-Att	20.12	54.74%	1.05%	0.00%	19.24	55.86%	1.38%	0.00%	32.02	56.82%	2.87%	2.29%
	Full-Ref	20.12	75.96%	3.14%	0.30%	19.24	86.64%	26.63%	0.38%	32.02	85.30%	36.68%	15.76%
Head-FT		Val-PPL	AUC	@FPR1%	@FPR0.01%	Val-PPL	AUC	@FPR1%	@FPR0.01%	Val-PPL	AUC	@FPR1%	@FPR0.01%
PreCurious	Basic	18.56	96.63%	21.71%	2.40%	17.69	98.77%	80.93%	24.49%	28.06	99.32%	74.79%	47.85%
	Stealthy	19.18	94.41%	18.86%	0.30%	18.20	95.35%	58.39%	19.50%	29.02	99.70%	87.39%	79.94%
Benign	Loss-Att	35.93	54.72%	1.20%	0.00%	30.57	52.97%	1.24%	0.00%	50.31	54.79%	3.44%	1.72%
	Full-Ref	35.93	57.26%	6.29%	0.45%	30.57	56.56%	0.02%	0.00%	50.31	68.18%	4.30%	2.29%
Full-FT		Val-PPL	AUC	@FPR1%	@FPR0.01%	Val-PPL	AUC	@FPR1%	@FPR0.01%	Val-PPL	AUC	@FPR1%	@FPR0.01%
PreCurious	Basic	16.68	96.49%	30.24%	1.95%	15.46	99.99%	100.00%	99.95%	22.31	99.99%	100.00%	99.43%
	Stealthy	16.84	96.17%	35.03%	2.10%	17.45	72.92%	7.56%	1.24%	23.07	99.97%	99.71%	97.99%
Benign	Loss-Att	18.49	62.95%	1.20%	0.00%	17.42	64.85%	1.81%	0.00%	27.67	66.79%	4.58%	2.87%
	Full-Ref	18.49	91.56%	14.22%	1.35%	17.42	98.93%	90.16%	73.04%	27.67	93.39%	66.48%	64.18%

**Table 2:** Membership inference evaluation on GPT-2 medium and GPT-2 large with AdapterFT $\left(E_{\text{ft}}=5,E_{\text{Pr}e}=3\right)$

Adapter-FT			Enron				PubMed				PTB		
GPT-2 Medium		Val-PPL	AUC	@FPR1%	@FPR0.01%	Val-PPL	AUC	@FPR1%	@FPR0.01%	Val-PPL	AUC	@FPR1%	@FPR0.01%
PreCurious	Basic	14.18	84.31%	6.29%	0.75%	13.01	96.48%	51.93%	2.38%	20.11	97.47%	67.05%	48.71%
Benign	Loss-Att	17.17	53.48%	1.20%	0.15%	14.82	54.68%	1.19%	0.00%	26.97	53.62%	1.72%	1.15%
	Full-Ref	17.17	58.12%	2.40%	0.75%	14.82	73.39%	9.89%	1.14%	26.97	62.81%	5.16%	2.58%
GPT-2 Large		Val-PPL	AUC	@FPR1%	@FPR0.01%	Val-PPL	AUC	@FPR1%	@FPR0.01%	Val-PPL	AUC	@FPR1%	@FPR0.01%
PreCurious	Basic	12.39	87.24%	29.34%	5.54%	11.64	98.25%	73.99%	0.05%	16.94	99.40%	97.99%	96.56%
Benign	Loss-Att	14.92	57.01%	1.05%	0.15%	12.82	59.47%	1.81%	0.00%	21.66	60.79%	3.15%	2.29%
	Full-Ref	14.92	62.55%	6.44%	2.25%	12.82	85.66%	24.68%	0.00%	21.66	78.78%	31.81%	24.07%

**Table 3:** Membership inference evaluation on GPT-2 with Adapter-FT w/o $\theta_{\text{ref}}\left(E_{\text{ft}}=20,E_{\text{pre}}=1\right)$

Dataset		Enron				PubMed				PTB		
Adapter-FT	Val-PPL	AUC	@FPR1%	@FPR0.01%	Val-PPL	AUC	@FPR1%	@FPR0.01%	Val-PPL	AUC	@FPR1%	@FPR0.01%
PreCurious-Accelerated	18.11	55.59%	1.20%	0.00%	16.08	56.78%	1.10%	0.00%	26.70	58.03%	3.73%	2.01%
PreCurious-Basic	18.17	55.34%	1.20%	0.00%	16.09	56.63%	1.19%	0.00%	26.54	57.25%	3.15%	1.72%
Benign	19.84	55.00%	1.05%	0.00%	18.71	56.04%	1.47%	0.00%	30.43	56.97%	2.58%	2.29%

**Table 4:** Stealthiness on crafted $\theta_{\text{pre}}$. The red cell denotes ‘suspicious’ and green cell indicates ‘evaded’.

Dataset	Released Model	$S_{\text{mia}}$	$S_{\text{mem}}$	$S_{\text{gap}}$
Enron	Benign	0.5130	0.0359	**−3.7130**
	Accelerated	**0.5008**	0.0255	-0.8853
	Basic	0.5054	0.0494	-0.8963
	Stealthy	0.5090	0.0479	-1.1640
	Lagging	**0.5008**	**0.0000**	12.9240
Pubmed	Benign	**0.5010**	0.0005	−0.0650
	Accelerated	0.5084	0.0029	−0.0940
	Basic	0.5071	0.0029	−0.0974
	Stealthy	0.5060	0.0024	−0.1105
	Lagging	0.5049	**0.0000**	**−1.2530**
Ptb	Benign	0.4834	0.0057	6.5190
	Accelerated	**0.4805**	0.0086	2.5140
	Basic	0.4819	0.0086	3.0630
	Stealthy	0.4816	0.0086	**2.3150**
	Lagging	0.5019	**0.0000**	3.8090

**Table 5:** MIA effectiveness under weight-decaying on Enron dataset with LoRA-FT (w/ weight decay factor 0.5).

Model Init.	AUC w/o $\theta_{\text{ref}}$	@0.01FPR	@0.1FPR	AUC	Tr-PPL	Val-PPL
Benign	54.37%	2.40%	38.32%	73.48%	20.18	20.19
PreCurious	**55.18%**	**15.57%**	**85.63%**	**92.70%**	**16.61**	**17.07**

**Table 6:** MIA effectiveness under DP fine-tuning defense on PTB dataset with Adapter-FT (ϵ=1).

Model Init.	Strategy	@0.01FPR	@0.1FPR	AUC	Val-PPL
Benign	Full-Ref	**1.72%**	10.03%	52.05%	68.61
PreCurious	Basic	0.86%	**14.04%**	**54.84%**	**25.94**

**Table 7:** Comparison with related works that manipulate integrity for privacy risk amplification. Manipulate: ○/ ◐ / 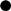 represents manipulating model parameters/model/training data; PEFT: ○/ ◐ / 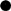 represents no/evaluated/evaluated and investigated. Case II: whether considering comparison cases when the fine-tuner applies early stopping. Stealthy: whether considering stealthiness control.

Method	Attacker’s Goal	Victim’s Task	Manipulate	Case II	Stealthy	PEFT
[7]	MIA	Discriminative	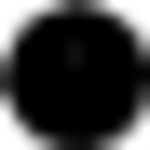	N/A	yes	○
[29]	Property inference	Discriminative	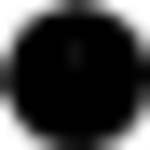	N/A	no	○
[44]	MIA+Extraction	Generative	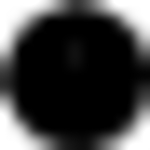	N/A	no	○
[43]	Property inference	Discriminative	○	N/A	yes	○
[11]	Reconstruction	Discriminative	◐	N/A	no	○
[47]	MIA	Generative	○	no	yes	◐
Ours	MIA+Extraction	Generative	○	yes	yes	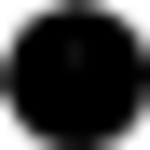

## CCS Concepts

• Security and privacy; • Computing methodologies → Artificial intelligence;

## A Experimental Setup and More Results

Our experiments were conducted on an Ubuntu 20.04.6 system with 8 NVIDIA Quadro RTX 8000 GPUs. The source code and other artifacts have been made available^2^.

**Table 8:** MIA effectiveness under DP-SGD defense on PTB dataset with AdapterFT.

ϵ	MIA metric	TPR@0.01FPR	TPR@0.1FPR	AUC	Val-PPL
0.05	Benign	**2.29%**	10.03%	51.99%	73.64
0.05	PreCurious	0.86%	**12.89%**	**53.53%**	**27.64**
0.5	Benign	**1.72%**	10.03%	52.03%	70.41
0.5	PreCurious	1.43%	**13.47%**	**55.09%**	**26.42**
1	Benign	**1.72%**	10.03%	52.05%	68.61
1	PreCurious	0.86%	**14.04%**	**54.84%**	**25.94**
2	Benign	**1.72%**	9.74%	52.01%	66.59
2	PreCurious	1.15%	**14.33%**	**54.58%**	**25.47**

**Table 9:** Untargeted $p_{\text{ext}}↑$ on PTB with Adapter-FT.

ϵ	Pre-trained model	Subsequence Length			
	(w/ or w/o Ref)	2	5	10	50
0.05	PreCurious w/ Ref	91.78%	57.85%	39.43%	18.10%
0.05	PreCurious w/o Ref	56.95%	49.65%	37.10%	19.80%
0.05	Benign w/ Ref	65.68%	39.20%	37.08%	20.94%
0.05	Benign w/o Ref	46.67%	41.34%	36.84%	18.33%
8	PreCurious w/ Ref	92.88%	58.81%	39.04%	18.67%
8	PreCurious w/o Ref	65.43%	57.67%	37.13%	19.92%
8	Benign w/ Ref	62.53%	39.21%	37.20%	21.32%
8	Benign w/o Ref	44.11%	39.20%	36.88%	18.84%

## References

[1] Accessed: 2023-12-07. Github. https://github.com.

[2] Accessed: 2023-12-07. Hugging Face Models. https://huggingface.co/models.

[3] Carlini Nicholas, Chien Steve, Nasr Milad, Song Shuang, Terzis Andreas, and Tramer Florian. 2022. Membership inference attacks from first principles. In 2022 IEEE Symposium on Security and Privacy (SP). IEEE, 1897–1914.

[4] Carlini Nicholas, Ippolito Daphne, Jagielski Matthew, Lee Katherine, Tramer Florian, and Zhang Chiyuan. 2022. Quantifying memorization across neural language models. arXiv preprint arXiv:2202.07646 (2022).

[5] Carlini Nicholas, Liu Chang, Erlingsson Úlfar, Kos Jernej, and Song Dawn. 2019. The secret sharer: Evaluating and testing unintended memorization in neural networks. In 28th USENIX Security Symposium (USENIX Security 19). 267–284.

[6] Carlini Nicholas, Tramer Florian, Wallace Eric, Jagielski Matthew, Herbert-Voss Ariel, Lee Katherine, Roberts Adam, Brown Tom, Song Dawn, Erlingsson Ulfar, 2021. Extracting training data from large language models. In 30th USENIX Security Symposium (USENIX Security 21). 2633–2650.

[7] Chen Yufei, Shen Chao, Shen Yun, Wang Cong, and Zhang Yang. 2022. Amplifying membership exposure via data poisoning. Advances in Neural Information Processing Systems 35 (2022), 29830–29844.

[8] Choquette-Choo Christopher A, Tramer Florian, Carlini Nicholas, and Papernot Nicolas. 2021. Label-only membership inference attacks. In International conference on machine learning. PMLR, 1964–1974.

[9] Cohan Arman, Dernoncourt Franck, Kim Doo Soon, Bui Trung, Kim Seokhwan, Chang Walter, and Goharian Nazli. 2018. A Discourse-Aware Attention Model for Abstractive Summarization of Long Documents. In Proceedings of the 2018 Conference of the North American Chapter of the Association for Computational Linguistics: Human Language Technologies, Volume 2 (Short Papers). Association for Computational Linguistics, New Orleans, Louisiana, 615–621. 10.18653/v1/N18-2097

[10] Donahue Jeff, Jia Yangqing, Vinyals Oriol, Hoffman Judy, Zhang Ning, Tzeng Eric, and Darrell Trevor. 2014. Decaf: A deep convolutional activation feature for generic visual recognition. In International conference on machine learning. PMLR, 647–655.

[11] Feng Shanglun and Tramèr Florian. 2024. Privacy Backdoors: Stealing Data with Corrupted Pretrained Models. arXiv preprint arXiv:2404.00473 (2024).

[12] He Junxian, Zhou Chunting, Ma Xuezhe, Berg-Kirkpatrick Taylor, and Neubig Graham. 2021. Towards a unified view of parameter-efficient transfer learning. arXiv preprint arXiv:2110.04366 (2021).

[13] Houlsby Neil, Giurgiu Andrei, Jastrzebski Stanislaw, Morrone Bruna, De Laroussilhe Quentin, Gesmundo Andrea, Attariyan Mona, and Gelly Sylvain. 2019. Parameter-efficient transfer learning for NLP. In International Conference on Machine Learning. PMLR, 2790–2799.

[14] Hu Edward J, Shen Yelong, Wallis Phillip, Allen-Zhu Zeyuan, Li Yuanzhi, Wang Shean, Wang Lu, and Chen Weizhu. 2021. Lora: Low-rank adaptation of large language models. arXiv preprint arXiv:2106.09685 (2021).

[15] Huang Kexin, Altosaar Jaan, and Ranganath Rajesh. 2019. Clinicalbert: Modeling clinical notes and predicting hospital readmission. arXiv preprint arXiv:1904.05342 (2019).

[16] Humphries Thomas, Oya Simon, Tulloch Lindsey, Rafuse Matthew, Goldberg Ian, Hengartner Urs, and Kerschbaum Florian. 2023. Investigating membership inference attacks under data dependencies. In 2023 IEEE 36th Computer Security Foundations Symposium (CSF). IEEE, 473–488.

[17] Jayaraman Bargav, Wang Lingxiao, Knipmeyer Katherine, Gu Quanquan, and Evans David. 2020. Revisiting membership inference under realistic assumptions. arXiv preprint arXiv:2005.10881 (2020).

[18] Jia Jinyuan, Liu Yupei, and Gong Neil Zhenqiang. 2022. Badencoder: Backdoor attacks to pre-trained encoders in self-supervised learning. In 2022 IEEE Symposium on Security and Privacy (SP). IEEE, 2043–2059.

[19] Johnson Alistair, Pollard Tom, Horng Steven, Celi Leo Anthony, and Mark Roger. 2023. MIMIC-IV-Note: Deidentified free-text clinical notes.

[20] Kandpal Nikhil, Wallace Eric, and Raffel Colin. 2022. Deduplicating training data mitigates privacy risks in language models. In International Conference on Machine Learning. PMLR, 10697–10707.

[21] Klimt Bryan and Yang Yiming. 2004. The Enron Corpus: A New Dataset for Email Classification Research. In Proceedings of the 15th European Conference on Machine Learning (Pisa, Italy) (ECML’04). Springer-Verlag, Berlin, Heidelberg, 217–226. 10.1007/978-3-540-30115-8_22

[22] Lee Jinhyuk, Yoon Wonjin, Kim Sungdong, Kim Donghyeon, Kim Sunkyu, So Chan Ho, and Kang Jaewoo. 2020. BioBERT: a pre-trained biomedical language representation model for biomedical text mining. Bioinformatics 36, 4 (2020), 1234–1240. 31501885 10.1093/bioinformatics/btz682 PMC7703786

[23] Lee Katherine, Ippolito Daphne, Nystrom Andrew, Zhang Chiyuan, Eck Douglas, Callison-Burch Chris, and Carlini Nicholas. 2021. Deduplicating training data makes language models better. arXiv preprint arXiv:2107.06499 (2021).

[24] Li Xuechen, Tramer Florian, Liang Percy, and Hashimoto Tatsunori. 2021. Large language models can be strong differentially private learners. arXiv preprint arXiv:2110.05679 (2021).

[25] Li Zheng and Zhang Yang. 2021. Membership leakage in label-only exposures. In Proceedings of the 2021 ACM SIGSAC Conference on Computer and Communications Security. 880–895.

[26] Lialin Vladislav, Deshpande Vijeta, and Rumshisky Anna. 2023. Scaling down to scale up: A guide to parameter-efficient fine-tuning. arXiv preprint arXiv:2303.15647 (2023).

[27] Long Yunhui, Bindschaedler Vincent, Wang Lei, Bu Diyue, Wang Xiaofeng, Tang Haixu, Gunter Carl A, and Chen Kai. 2018. Understanding membership inferences on well-generalized learning models. arXiv preprint arXiv:1802.04889 (2018).

[28] Long Yunhui, Wang Lei, Bu Diyue, Bindschaedler Vincent, Wang Xiaofeng, Tang Haixu, Gunter Carl A, and Chen Kai. 2020. A pragmatic approach to membership inferences on machine learning models. In 2020 IEEE European Symposium on Security and Privacy (EuroS&P). IEEE, 521–534.

[29] Mahloujifar Saeed, Ghosh Esha, and Chase Melissa. 2022. Property inference from poisoning. In 2022 IEEE Symposium on Security and Privacy (SP). IEEE, 1120–1137.

[30] Maini Pratyush, Mozer Michael C, Sedghi Hanie, Lipton Zachary C, Kolter J Zico, and Zhang Chiyuan. 2023. Can neural network memorization be localized? arXiv preprint arXiv:2307.09542 (2023).

[31] Marcus Mitchell P., Santorini Beatrice, and Marcinkiewicz Mary Ann. 1993. Building a Large Annotated Corpus of English: The Penn Treebank. Computational Linguistics 19, 2 (1993), 313–330. https://www.aclweb.org/anthology/J93-2004

[32] Mattern Justus, Mireshghallah Fatemehsadat, Jin Zhijing, Schölkopf Bernhard, Sachan Mrinmaya, and Berg-Kirkpatrick Taylor. 2023. Membership Inference Attacks against Language Models via Neighbourhood Comparison. arXiv preprint arXiv:2305.18462 (2023).

[33] Mireshghallah Fatemehsadat, Goyal Kartik, Uniyal Archit, Berg-Kirkpatrick Taylor, and Shokri Reza. 2022. Quantifying privacy risks of masked language models using membership inference attacks. arXiv preprint arXiv:2203.03929 (2022).

[34] Mireshghallah Fatemehsadat, Uniyal Archit, Wang Tianhao, Evans David, and Berg-Kirkpatrick Taylor. 2022. Memorization in nlp fine-tuning methods. arXiv preprint arXiv:2205.12506 (2022).

[35] Nasr Milad, Shokri Reza, and Houmansadr Amir. 2019. Comprehensive privacy analysis of deep learning: Passive and active white-box inference attacks against centralized and federated learning. In 2019 IEEE symposium on security and privacy (SP). IEEE, 739–753.

[36] O’hagan A and Leonard Tom. 1976. Bayes estimation subject to uncertainty about parameter constraints. Biometrika 63, 1 (1976), 201–203.

[37] Pfeiffer Jonas, Rücklé Andreas, Poth Clifton, Kamath Aishwarya, Vulić Ivan, Ruder Sebastian, Cho Kyunghyun, and Gurevych Iryna. 2020. Adapterhub: A framework for adapting transformers. arXiv preprint arXiv:2007.07779 (2020).

[38] Radford Alec, Wu Jeffrey, Child Rewon, Luan David, Amodei Dario, Sutskever Ilya, 2019. Language models are unsupervised multitask learners. OpenAI blog 1, 8 (2019), 9.

[39] Sablayrolles Alexandre, Douze Matthijs, Schmid Cordelia, Ollivier Yann, and Jégou Hervé. 2019. White-box vs black-box: Bayes optimal strategies for membership inference. In International Conference on Machine Learning. PMLR, 5558–5567.

[40] Salem Ahmed, Cherubin Giovanni, Evans David, Köpf Boris, Paverd Andrew, Suri Anshuman, Tople Shruti, and Zanella-Béguelin Santiago. 2023. SoK: Let the privacy games begin! A unified treatment of data inference privacy in machine learning. In 2023 IEEE Symposium on Security and Privacy (SP). IEEE, 327–345.

[41] Shokri Reza, Stronati Marco, Song Congzheng, and Shmatikov Vitaly. 2017. Membership inference attacks against machine learning models. In 2017 IEEE symposium on security and privacy (SP). IEEE, 3–18.

[42] Srivastava Nitish, Hinton Geoffrey, Krizhevsky Alex, Sutskever Ilya, and Salakhutdinov Ruslan. 2014. Dropout: a simple way to prevent neural networks from overfitting. The journal of machine learning research 15, 1 (2014), 1929–1958.

[43] Tian Yulong, Suya Fnu, Suri Anshuman, Xu Fengyuan, and Evans David. 2023. Manipulating Transfer Learning for Property Inference. In Proceedings of the IEEE/CVF Conference on Computer Vision and Pattern Recognition. 15975–15984.

[44] Tramèr Florian, Shokri Reza, Joaquin Ayrton San, Le Hoang, Jagielski Matthew, Hong Sanghyun, and Carlini Nicholas. 2022. Truth serum: Poisoning machine learning models to reveal their secrets. In Proceedings of the 2022 ACM SIGSAC Conference on Computer and Communications Security. 2779–2792.

[45] Watson Lauren, Guo Chuan, Cormode Graham, and Sablayrolles Alex. 2021. On the importance of difficulty calibration in membership inference attacks. arXiv preprint arXiv:2111.08440 (2021).

[46] Wen Rui, Wang Tianhao, Backes Michael, Zhang Yang, and Salem Ahmed. 2023. Last One Standing: A Comparative Analysis of Security and Privacy of Soft Prompt Tuning, LoRA, and In-Context Learning. arXiv preprint arXiv:2310.11397 (2023).

[47] Wen Yuxin, Marchyok Leo, Hong Sanghyun, Geiping Jonas, Goldstein Tom, and Carlini Nicholas. 2024. Privacy Backdoors: Enhancing Membership Inference through Poisoning Pre-trained Models. arXiv preprint arXiv:2404.01231 (2024).

[48] Ye Jiayuan, Maddi Aadyaa, Murakonda Sasi Kumar, Bindschaedler Vincent, and Shokri Reza. 2022. Enhanced membership inference attacks against machine learning models. In Proceedings of the 2022 ACM SIGSAC Conference on Computer and Communications Security. 3093–3106.

[49] Ye Jiayuan, Zhu Zhenyu, Liu Fanghui, Shokri Reza, and Cevher Volkan. 2023. Initialization Matters: Privacy-Utility Analysis of Overparameterized Neural Networks. arXiv preprint arXiv:2310.20579 (2023).

[50] Yeom Samuel, Giacomelli Irene, Fredrikson Matt, and Jha Somesh. 2018. Privacy risk in machine learning: Analyzing the connection to overfitting. In 2018 IEEE 31st computer security foundations symposium (CSF). IEEE, 268–282.

[51] Yu Da, Naik Saurabh, Backurs Arturs, Gopi Sivakanth, Inan Huseyin A, Kamath Gautam, Kulkarni Janardhan, Yin Tat Lee Andre Manoel, Wutschitz Lukas, 2021. Differentially private fine-tuning of language models. arXiv preprint arXiv:2110.06500 (2021).

[52] Zaken Elad Ben, Ravfogel Shauli, and Goldberg Yoav. 2021. Bitfit: Simple parameter-efficient fine-tuning for transformer-based masked language-models. arXiv preprint arXiv:2106.10199 (2021).

[53] Zhang Xinyang, Zhang Zheng, Ji Shouling, and Wang Ting. 2021. Trojaning language models for fun and profit. In 2021 IEEE European Symposium on Security and Privacy (EuroS&P). IEEE, 179–197.

